# Antenna arrangement and energy-transfer pathways of PSI–LHCI from the moss *Physcomitrella patens*

**DOI:** 10.1038/s41421-021-00242-9

**Published:** 2021-02-16

**Authors:** Qiujing Yan, Liang Zhao, Wenda Wang, Xiong Pi, Guangye Han, Jie Wang, Lingpeng Cheng, Yi-Kun He, Tingyun Kuang, Xiaochun Qin, Sen-Fang Sui, Jian-Ren Shen

**Affiliations:** 1 Photosynthesis Research Center, Key Laboratory of Photobiology, Institute of Botany, Chinese Academy of Sciences, Beijing, 100093 China; 2 University of Chinese Academy of Sciences, Beijing, 100049 China; 3 State Key Laboratory of Membrane Biology, Beijing Advanced Innovation Center for Structural Biology & Frontier Research Center for Biological Structure, School of Life Sciences, Tsinghua University, Beijing, 100084 China; 4 College of Life Sciences, Department of Chemistry, Capital Normal University, Beijing, 100048 China; 5 School of Biological Science and Technology, University of Jinan, Jinan, Shandong, 250022 China; 6 Department of Biology, Southern University of Science and Technology, Shenzhen, Guangdong, 518055 China; 7 Research Institute for Interdisciplinary Science, and Graduate School of Natural Science and Technology, Okayama University, Okayama, 700-8530 Japan

**Keywords:** Cryoelectron microscopy, Plant molecular biology

## Abstract

Plants harvest light energy utilized for photosynthesis by light-harvesting complex I and II (LHCI and LHCII) surrounding photosystem I and II (PSI and PSII), respectively. During the evolution of green plants, moss is at an evolutionarily intermediate position from aquatic photosynthetic organisms to land plants, being the first photosynthetic organisms that landed. Here, we report the structure of the PSI–LHCI supercomplex from the moss *Physcomitrella patens* (*Pp*) at 3.23 Å resolution solved by cryo-electron microscopy. Our structure revealed that four Lhca subunits are associated with the PSI core in an order of Lhca1–Lhca5–Lhca2–Lhca3. This number is much decreased from 8 to 10, the number of subunits in most green algal PSI–LHCI, but the same as those of land plants. Although *Pp* PSI–LHCI has a similar structure as PSI–LHCI of land plants, it has Lhca5, instead of Lhca4, in the second position of Lhca, and several differences were found in the arrangement of chlorophylls among green algal, moss, and land plant PSI–LHCI. One chlorophyll, PsaF–Chl 305, which is found in the moss PSI–LHCI, is located at the gap region between the two middle Lhca subunits and the PSI core, and therefore may make the excitation energy transfer from LHCI to the core more efficient than that of land plants. On the other hand, energy-transfer paths at the two side Lhca subunits are relatively conserved. These results provide a structural basis for unravelling the mechanisms of light-energy harvesting and transfer in the moss PSI–LHCI, as well as important clues on the changes of PSI–LHCI after landing.

## Introduction

Photosynthesis harvests and converts light energy from the sun into chemical energy that sustains almost all life activities on the earth. Four complexes, namely, photosystem I (PSI), photosystem II (PSII), cytochrome *b6f*, and ATP synthase, located on the thylakoid membrane of various organisms, are responsible for capturing and converting light energy into ATP and NADPH for further carbon dioxide fixation. Among them, PSI uses the peripheral antennas to absorb light and transfer the excitation energy to the PSI core complex consisting of core antennas and the reaction center P700, thereby driving charge separation and transmembrane electron transfer. Since PSI is one of the most efficient light-energy capture and energy conversion devices in nature^1,2^, solving the structure of PSI is of great importance for understanding mechanisms of photosynthetic light reactions.

PSI core complex from prokaryotic cyanobacteria usually exists as a trimer^3^, which binds membrane-extrinsic, soluble phycobilisome as the peripheral antenna^4,5^. In contrast, PSI cores from eukaryotic algae and higher plants exist as a monomer, which is associated with various membrane-spanning light-harvesting complex I (LHCI), forming PSI–LHCI supercomplexes. The structures of PSI–LHCI supercomplexes have been solved from various eukaryotic organisms such as red algae^6^, diatoms^7,8^, green algae^9–11^, and land plants^12–14^. These studies showed that the PSI core is relatively conserved during more than 2.5 billion years of evolution, whereas there is a large diversity in the pigment and protein compositions of LHCI from different organisms^15^. The PSI–LHCI structure from land plants, *Pisum sativum* (*P*. *sativum*, *Ps*) and *Zea mays* (*Z*. *mays*, *Zm*), showed that four LHCI proteins (Lhca1 to Lhca4) are assembled into a crescent-shaped LHCI belt surrounding the core complex^12–14^. In contrast, the PSI–LHCI structure from two different types of green algae, a microalga *Chlamydomonas reinhardtii* (*C*. *reinhardtii*, *Cr*) and a macroalga *Bryopsis corticulans* (*B*. *corticulans*, *Bc*), revealed the association of up to ten Lhca subunits around the PSI core^9–11^. The distinct structural differences between aquatic green algae and land plants may be a result of adaptation to different light environments that different organisms experience^15^.

Bryophytes (liverworts, mosses, and hornworts) are derived from the ancestor of early land plants^16^, and are the first group of plants that shifted from aquatic to terrestrial environments. Therefore, the organization of PSI–LHCI of bryophytes is of great interest for understanding the transition of the photosynthetic apparatus from aquatic to terrestrial environments during the evolution of green plants. The structures of two types of PSI complexes with different antenna sizes have been reported from the moss *Physcomitrella patens* (*P*. *patens*, *Pp*)^17–20^; however, neither was at a resolution high enough to reveal the detailed information about the antenna organization and pigment arrangement.

In this study, we purified the PSI–LHCI supercomplex from the moss *P*. *patens*, and solved its structure at an overall resolution of 3.23 Å using single-particle cryo-electron microscopy (cryo-EM). The structure reveals the association of four Lhcas to the PSI core, which is similar to that in higher plant PSI–LHCI, but with Lhca5 replacing Lhca4, and sheds light on the mechanisms of light-energy harvesting and transfer in the PSI–LHCI from bryophytes. These results also provide important clues to the evolutionary changes that have occurred in PSI–LHCI from aquatic algae to land plants.

## Results

### Purification and characterization of *P. patens* PSI–LHCI

Photosynthetic pigment-protein complexes were isolated from *P*. *patens* by anion-exchange chromatography and sucrose density gradient ultracentrifugation (see “Materials and methods”; Supplementary Fig. S1a, b), and a dark green band in the lower part of the gradient was identified as PSI–LHCI by SDS-PAGE (Supplementary Fig. S1b, c). All bands resolved in the gel were analyzed by mass spectrometry, among which four bands were identified as Lhca1, Lhca2, Lhca3, and Lhca5, respectively (Supplementary Fig. S1c). The room temperature absorption spectrum of *P*. *patens* PSI–LHCI (*Pp* PSI–LHCI) shows two peaks at 436 and 680 nm, respectively (Supplementary Fig. S1d), and its low-temperature (77 K) fluorescence emission spectrum shows a major peak at 727 nm (Supplementary Fig. S1e). It is notable that the fluorescence emission peak of *Pp* PSI–LHCI is red-shifted as compared with the peak of PSI–LHCI (709 nm) from a green algae *B*. *corticulans*, and is blue-shifted as compared with the peak of PSI–LHCI (735 nm) from a land plant *P*. *sativum* (Supplementary Fig. S1e). The long wavelength fluorescence emission is an important feature for PSI, which arose from several chlorophyll (Chl) molecules, called red Chls, mainly bound to Lhca subunits^1,21^. The differences in the wavelength of fluorescence emission among the three types of PSI–LHCI suggest different micro-environments of the red Chls among the different organisms, which may be a result of adaption to the different light environments.

To reveal the detailed structure of *Pp* PSI–LHCI and provide a basis for further understanding its relationship with energy transfer and dissipation processes, we determined the structure by cryo-EM at a resolution of 3.23 Å (Supplementary Figs. S2, S3, and Table S1). We describe the structural features of the *Pp* PSI–LHCI supercomplex and its functional implications as follows.

### Overall structure

**Fig. 1:**
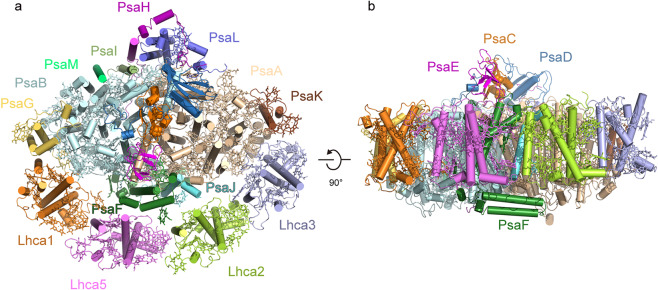
Overall structure of the PSI–LHCI supercomplex from *P*. *patens*. **a** View from the stromal side. **b** Side view along the membrane plane. Color codes: PsaA, wheet; PsaB, palecyan; PsaC, lightorange; PsaD, skyblue; PsaE, magenta; PsaF, forest; PsaG, yelloworange; PsaH, purple; PsaI, splitpea; PsaJ, cyan; PsaK, brown; PsaL, slate; PsaM, green; Lhca1, orange; Lhca5, violet; Lhca2, limon; Lhca3, lightblue.

The overall structure of the *Pp* PSI–LHCI is similar to that of land plants reported previously^12–14^, with a core complex composed of ten transmembrane subunits (PsaA, PsaB, PsaF, PsaG, PsaH, PsaI, PsaJ, PsaK, PsaL, and PsaM) and three extrinsic subunits (PsaC, PsaD, and PsaE) on the stromal side, and peripheral LHCI composed of four Lhca subunits surrounding the core at the PsaG–PsaF–PsaJ–PsaK side (Fig. 1; Supplementary Fig. S4). The four Lhca subunits can be assigned as Lhca1, Lhca5, Lhca2, and Lhca3 from PsaG to PsaK along the PsaG–PsaF–PsaJ–PsaK side, which is different from the order of Lhca1, Lhca4, Lhca2, and Lhca3 in *Ps* PSI–LHCI^12,13^ and *Zm* PSI–LHCI^14^.

There are many abundant *lhc* genes coding for Lhc proteins in the *P*. *patens* genome^22,23^. For example, *Pp* Lhca2 has five isoforms, both *Pp* Lhca1 and *Pp* Lhca3 has four isoforms, and only *Pp* Lhca5 has one; in contrast, *Arabidopsis thaliana* (*A*. *thaliana*, *At*) contains only one genetic isoform for each Lhca subunit (see “Materials and methods”; Supplementary Fig. S5). Considering the high sequence similarity for each group of the Lhca isoforms, the question is which isoform is selected for building the *Pp* PSI–LHCI, or can some of the Lhca positions be occupied by mixed isoforms. According to the cryo-EM map, the first and third Lhca positions were confirmed to be Lhca1 (xp_024393004.1) (Supplementary Fig. S6) and Lhca2 (xp_024386885.1) (Supplementary Fig. S7), respectively; the second position is Lhca5 which has only one form, whereas the fourth Lhca position (Supplementary Fig. S8) could not be distinguished among the Lhca3 isoforms.

Sequence alignment of Lhca subunits shows high similarity between the pairs of *Pp* Lhca1 and *At* Lhca1, *Pp* Lhca2 and *At* Lhca2, and *Pp* Lhca3 and *At* Lhca3 (Supplementary Fig. S9), whereas amino acid sequence of *Pp* Lhca5 shows higher similarity to *At* Lhca5 than to *At* Lhca4. Thus, the second Lhca position shows the main difference between *Pp* PSI–LHCI and PSI–LHCI of the land plants. There are only four Lhca genes in *P*. *patens*, and the gene homologous to lhca4 is not found in the *P*. *patens* genome^18,24^.

In addition to the protein subunits, we found 156 Chl molecules (144 Chls *a*; 12 Chls *b*), 34 carotenoid (Car) molecules (26 *β*-carotenes (BCRs); 4 violaxanthins, 4 luteins (LUTs)), and 10 lipids (6 phosphatidyl-glycerol, 1 digalactosyl diacylglycerol, and 3 monogalactosyl-diglyceride), in the *Pp* PSI–LHCI supercomplex (Supplementary Table S2).

### Structural features of the moss PSI core complex

Among the 13 core subunits in the *Pp* PSI–LHCI, ten (PsaA to PsaF and PsaI to PsaL) are conserved in all reported PSI structures from cyanobacteria to land plants. The subunits PsaA, PsaB, PsaC, PsaD, and PsaE provide the most fundamental protein basis for the PSI electron transfer reaction, which is consistent with their extremely conserved protein structures in the evolutionary process, indicating that the process of electron transfer is basically conserved in different species. Despite the high conservation of the PSI core, some variations are found in the subunit composition among different organisms and they are summarized in Supplementary Table S3.

The *Pp* PSI–LHCI lacks the subunit PsaO, although its gene exists in the moss genome, which suggests that it was lost during preparation due to its loose association with the PSI. PsaO has been resolved in the cryo-EM structures of a red algal PSI–LHCI^6^ and *Zm* PSI–LHCI–LHCII^14^, as well as a green alga *Dunaliella salina*^25^, but was not resolved in some structures of green algal^9–11^ and *Ps* PSI–LHCI^12,13^, suggesting that this subunit may bound loosely to the PSI core complex. *Pp* PSI–LHCI also does not contain PsaN because its gene is absent in the genome^24^, although the subunit exists in some green algae and land plants.

The *Pp* PSI–LHCI contains PsaM, which is the same as the PSI core from cyanobacteria^3^, red algae^6,26^, a green alga *B. corticulans*^9^ but absent in the green alga *C. reinhardtii*^10,11^. In the red algae and green alga *B. corticulans*, PsaM, together with the subunits PsaI and PsaB, promotes the functional association of an extra antenna dimer to the core complex^6,9^. By contrast, PsaM was absent in the PSI–LHCI supercomplexes of higher plants (*P*. *sativum* and *Z*. *mays*)^12–14^, where the extra antenna dimer was also absent. These results indicated that PsaM might be necessary for binding of the extra Lhca dimer. It was intriguing to note that the *Pp* PSI–LHCI contains the PsaM subunit but not the extra Lhca dimer (Fig. 1). In addition, PsaM was absent in another green alga *C. reinhardtii* where the extra Lhca dimer is associated with the PSI core. These may suggest another role of PsaM in the green algae and mosses not related with the association of the extra Lhca dimer.

PsaG can be variant during evolution. Red algal PSI–LHCI had not evolved PsaG, so that it contained only three Lhcas and the first Lhca position is empty^6^. By contrast, the *Pp* PSI–LHCI contains PsaG, making the first Lhca position being occupied, which is consist with the reported PSI–LHCI structures from green algae and land plants. This structure strengthened the viewpoint that PsaG has a critical role in binding one Lhca at the first Lhca position.

**Fig. 2:**
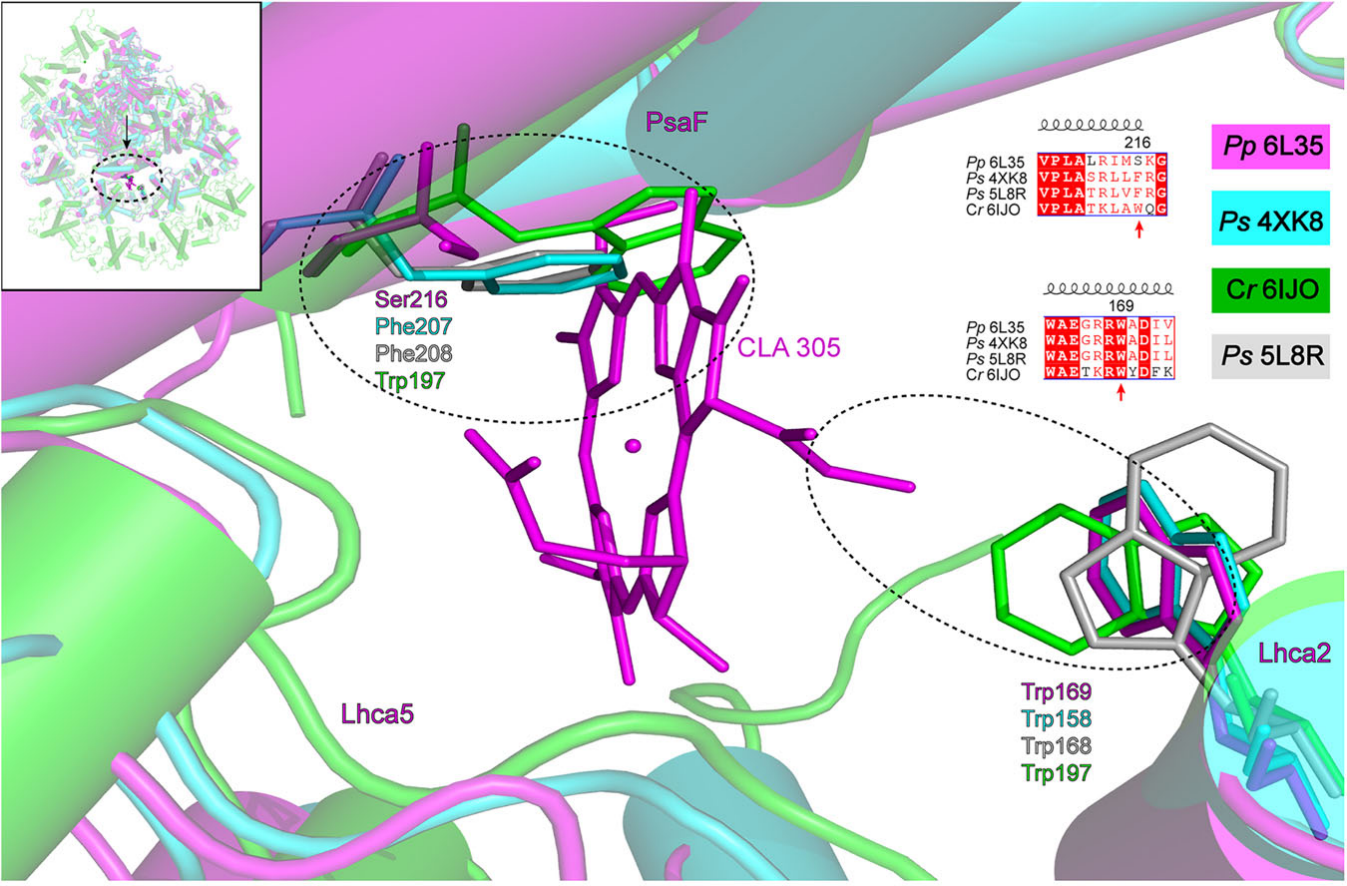
Structural comparison of PsaF among *P*. *patens*, *P*. *sativum*, and *C*. *reinhardtii*. CLA 305 clamped by PsaF, Lhca5, and Lhca2 in *P*. *patens* are shown as lines and labeled. The surrounding amino acid residues of Ser_216_ and Trp_169_ and corresponding residues in *P*. *sativum* and *C*. *reinhardtii* are shown and labeled. Color codes and PDB ID codes: *P*. *patens*, purple, 6L35; *C*. *reinhardtii*, green, 6IJO; *P*. *sativum*, cyan and gray, 4XK8 and 5L8R, respectively.

A Chl molecule (Chl *a*305) was found to bind to PsaF in the gap region between PsaF, Lhca5, and Lhca2 in the *Pp* PSI core (Fig. 1; Supplementary Figs. S10a and S11). This Chl was not found in any other PSI structures reported so far. To reveal the possible reason for binding of Chl *a*305, its surrounding environment was compared between *P*. *patens* and other organisms. Sequence alignment shows that the side chain of Ser_216_ of *Pp* PsaF was shorter than the side chain of Phe_207_ (PDB 4XK8) of *Ps* PsaF and Trp_197_ of *Cr* PsaF, which may create an additional space for binding of the Chl; whereas at the other side of the gap region Trp_169_ of Lhca2 is the same for different organisms (Fig. 2). Structural comparisons show that a large number of lipids bind to the gap region in the crystal structure of *Ps* PSI–LHCI, while less lipids were resolved in the *Pp* PSI–LHCI structure (Supplementary Fig. S11), and higher resolution may be needed to resolve lipids in the gap region. As no residue was found to coordinate Chl *a*305, it was tentatively speculated that some lipid molecules unresolved in the structure and/or water molecules would coordinate the Chl molecule. Similarly, some lipid molecules may be crucial for the stability of BCR103 clipped between PsaF and PsaJ near the lumenal side (Supplementary Figs. S10b and S11d). Effects of Chl *a*305 on the excitation energy-transfer (EET) pathways are discussed in detail in the following sections.

### Arrangement of Chls and carotenoids in LHCI

**Fig. 3:**
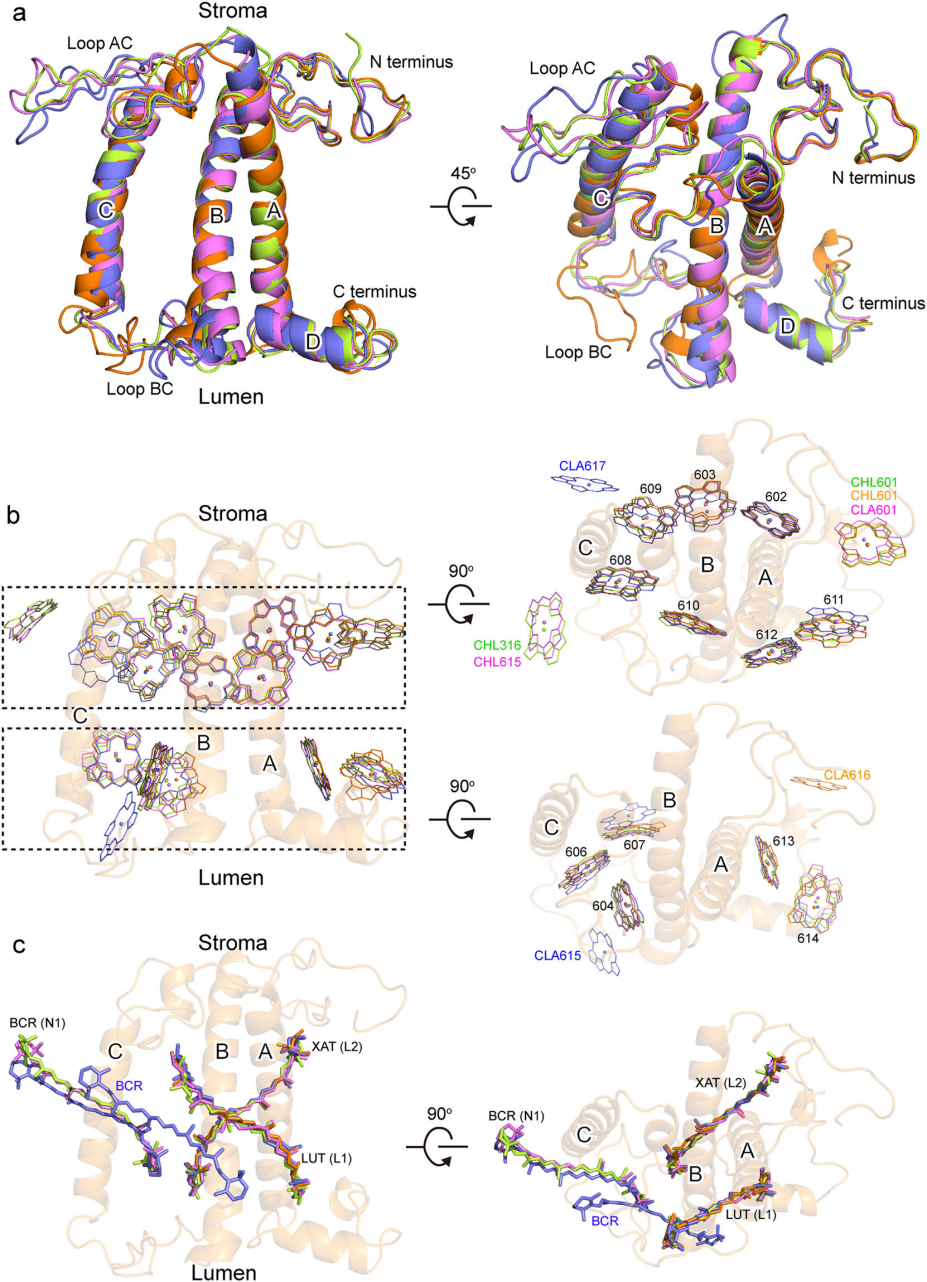
Comparison of the structures of the four Lhca subunits. **a** Superposition of the four Lhca apoprotein structures. **b** Superposition of Chl molecules from the four Lhca subunits, with the structure of the Lhca1 apoprotein shown in transparent orange in panels **b** and **c**. **c** Superposition of Car molecules from the four Lhca subunits. The color codes for the four Lhca subunits are the same as in Fig. 1, and those of pigments are the same as the Lhca subunits they are associated with. Special Chls mentioned in the text are labeled.

All Lhca subunits of *Pp* PSI–LHCI have similar structures as those of typical Lhc protein family, which are composed of three major transmembrane (TM) helices A, B, and C, an amphipathic helix D at the lumenal side, and two loop regions (loop AC and loop BC) connecting helix C with helix A or helix B (Fig. 3a). The structures of these Lhca subunits are also similar to the corresponding Lhca subunits from *P*. *sativum*^9–11^, suggesting that the interactions between each Lhca with the core are relatively conserved during evolution.

**Fig. 4:**
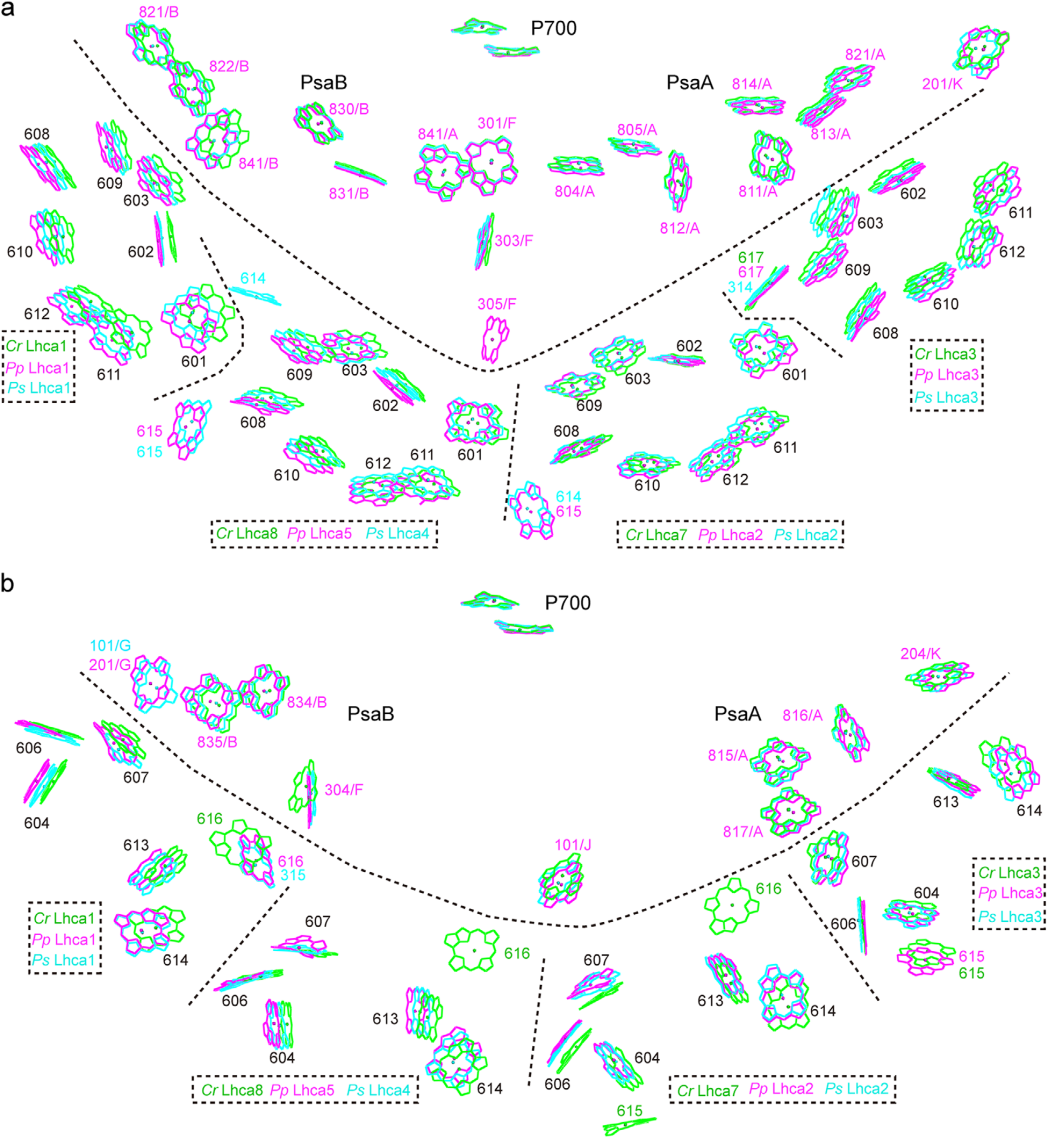
Comparison of the Chl arrangement in LHCI from a moss *P*. *patens*, a green alga *C*. *reinhardtii*, and a lant plant *P.sativum*. Part of the core antenna Chls near LHCI and reaction center P700 were also shown. **a** Chl arrangement at the stromal layer. **b** Chl arrangement at the lumenal layer. Chls are labeled in the same color as the Lhca subunits they are associated with. Color codes and PDB ID codes: *P*. *patens*, violet, 6L35; *C*. *reinhardtii*, green, 6IJO; *P*. *sativum*, cyan, 4XK8.

Each *Pp* Lhca subunit binds 14 Chl molecules, among which seven Chl molecules (Chl 602, 603, 608, 609, 610, 611, 612) are common ones located near the stromal surface, and five Chl molecules (Chl 604, 606, 607, 613, 614) are common ones located near the lumenal surface (Figs. 3 and 4). However, there are 1–2 Chls that are different among each Lhca, probably reflecting their specific roles in each Lhca. In the first Lhca position, Lhca1 binds a Chl *a*616 at the interface between its C-terminus and the core complex at the lumenal side, and this special Chl molecule is conserved from green algae to land plants (Fig. 3b), suggesting an important role of this Chl in all green plants. In the second and third Lhca positions, both Lhca5 and Lhca2 bind a Chl *b* molecule (Chl *b*615 of Lhca5 and Chl *b*316 of Lhca2) in the interface between the helix C and adjacent Lhca at the stromal side, which is the same as the corresponding Lhcas in *Ps* PSI–LHCI and suggesting the role of this Chl in interacting with the adjacent Lhca subunit. In the fourth Lhca position, Lhca3 binds a Chl *a*617 at the interface between its helix C and the core at the stromal side, a Chl that is also conserved from green algae to land plants (Fig. 3b). However, Chl *a*607 in Lhca3 of *Ps* PSI–LHCI is closer to the core complex than Chl *b*607 from other Lhca subunits, which is a unique feature of *Ps* PSI–LHCI. In addition, Lhca3 binds a Chl *b*615 at the lumenal loop BC region, which has been found to bind to Lhca3 from green algae^9–11^ but not from land plants *P*. *sativum* and *Z*. *mays*^12–14^. Chl 601 is found to bind to Lhca1, Lhca5/4, Lhca2 but not to Lhca3 (Fig. 4).

Lhca1 binds two Cars, Lhca2 and Lhca5 bind three Cars each, and Lhca3 binds four Cars (Supplementary Table S2). The number and binding sites of these Cars are largely conserved in green algal and higher plant PSI–LHCI. However, the green algal PSI–LHCI has one more Car in each position of Lhca1 and Lhca2, and the higher plant PSI–LHCI has two more Cars in the position of Lhca1 but lacks one Car in the position of Lhca3 (Supplementary Fig. S12). These features reflect the changes of Car binding in different organisms, which may affect the EET and quenching pathways.

### Assembly of LHCI belt

**Fig. 5:**
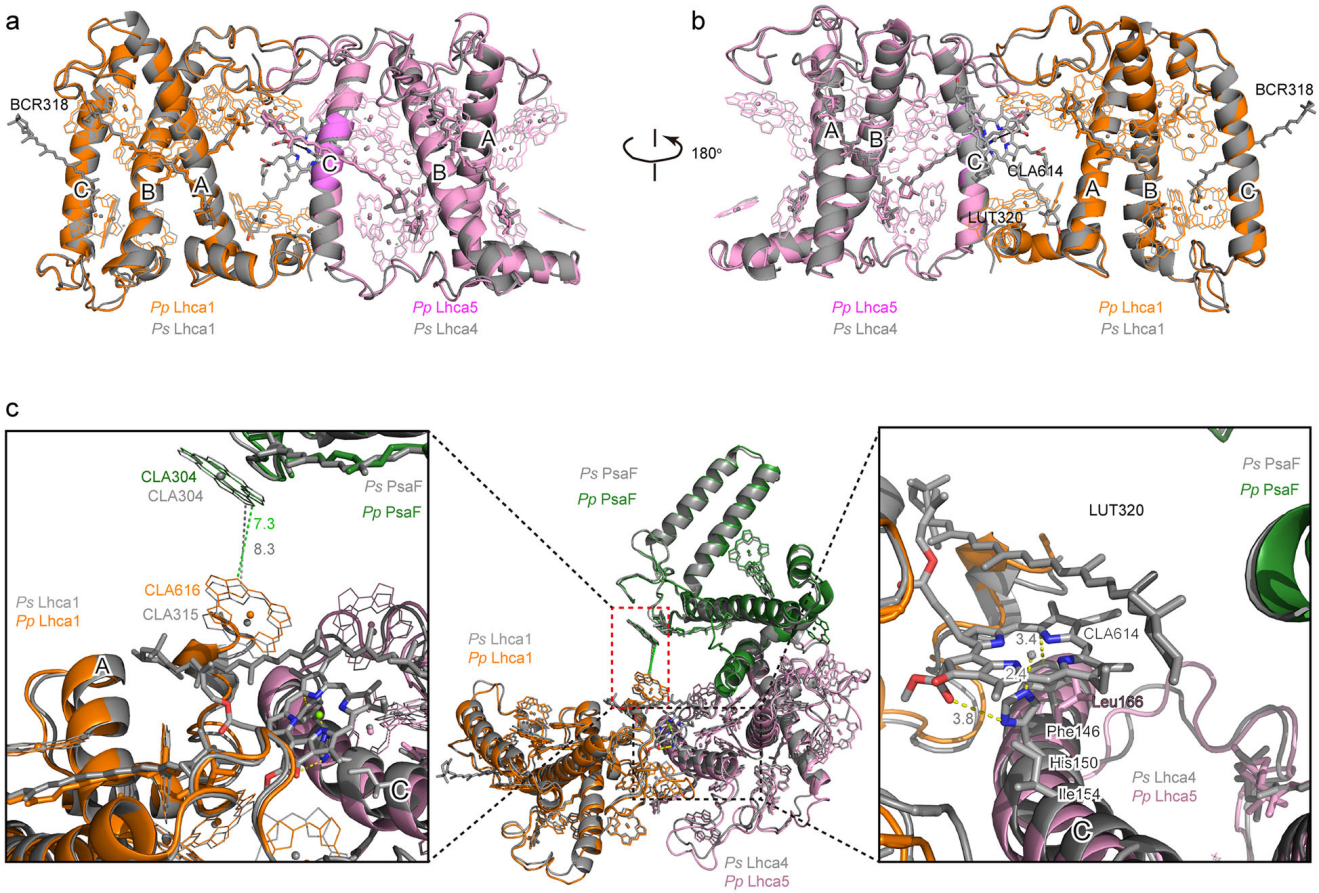
Structural comparison of Lhca1–Lhca5 dimer from *P*. *patens* with Lhca1–Lhca4 dimer from *P. sativum*. **a** Superposition of *Pp* Lhca1–Lhca5 with *Ps* Lhca1–Lhca4 viewed along the membrane from the Lhcas side to the PSI core side. **b** View along the membrane from the PSI core side to the Lhcas side. **c** Structure of Lhca1–Lhca5–PsaF from *P*. *patens*, showing the interactions between Lhca1–Lhca5 dimer and PsaF and the comparison with the corresponding part from *P*. *sativum*. Color codes and PDB ID codes: *P*. *patens*, Lhca1, orange; Lhca5, violet; Lhca2, limon; Lhca3, lightblue; 6L35; *P*. *sativum*, all subunits in gray, 4XK8.

In the structure of higher plant PSI–LHCI, LHCI is assembled as the dimer of dimers (Lhca1–Lhca4, Lhca2–Lhca3) and the interaction between Lhca1 and Lhca4 is the strongest among interactions between adjacent Lhca subunits^12,13^. Comparison between the *Ps* PSI–LHCI and *Pp* PSI–LHCI shows that the *Ps* Lhca1–Lhca4 dimer binds one Chl molecule (Chl *a*614) and one Car molecule (LUT320) located at the interface between Lhca1 and Lhca4, whereas they are absent in the *Pp* Lhca1–Lhca5 dimer (Fig. 5). Chl *a*614 is an important linking Chl for EET between Lhca1 and Lhca4, and LUT320 increases the interaction between the two Lhca subunits^12,13^, in the higher plant PSI–LHCI. The distinct difference arises from one amino acid modification happened in helix C of *Pp* Lhca5. In *Ps* Lhca4, His_150_ not only provides Chl *a*614 with a central Mg atom coordination, but also forms a hydrogen bond with the ester group of the ring E of Chl *a*614, and the surrounding hydrophobic Phe_146_ and Ile_154_ residues could further stabilize Chl *a*614 (Fig. 5c). Chl *a*614 may in turn enhance the binding of LUT320 to the interfacial region between Lhca1 and Lhca4 by providing a hydrophobic environment. However, the corresponding residue is changed to Gly_170_ in Lhca5 of *P*. *paten* (Supplementary Fig. S9g), which is not able to coordinate a Chl, and this further decreased the ability of binding an additional Car. Without the two connecting pigments, the interaction between Lhca1 and Lhca5 in *P*. *paten* is much weakened, and the EET pathways within the *Pp* PSI–LHCI supercomplex can be changed significantly when compared with the land plant *P*. *sativum*. The edge-to-edge distance between a linking Chl, Chl *a*616 of *Pp* Lhca1 or Chl *a*315 of *Ps* Lhca1, located at the lumenal side, to Chl *a*304/PsaF is changed from 7.3 Å in *P*. *patens* to 8.3 Å in *P*. *sativum* (Fig. 5c). However, the interaction between the second and the third Lhca is almost the same, no matter the second position is occupied by Lhca4 or Lhca5 (Supplementary Fig. S13).

**Fig. 6:**
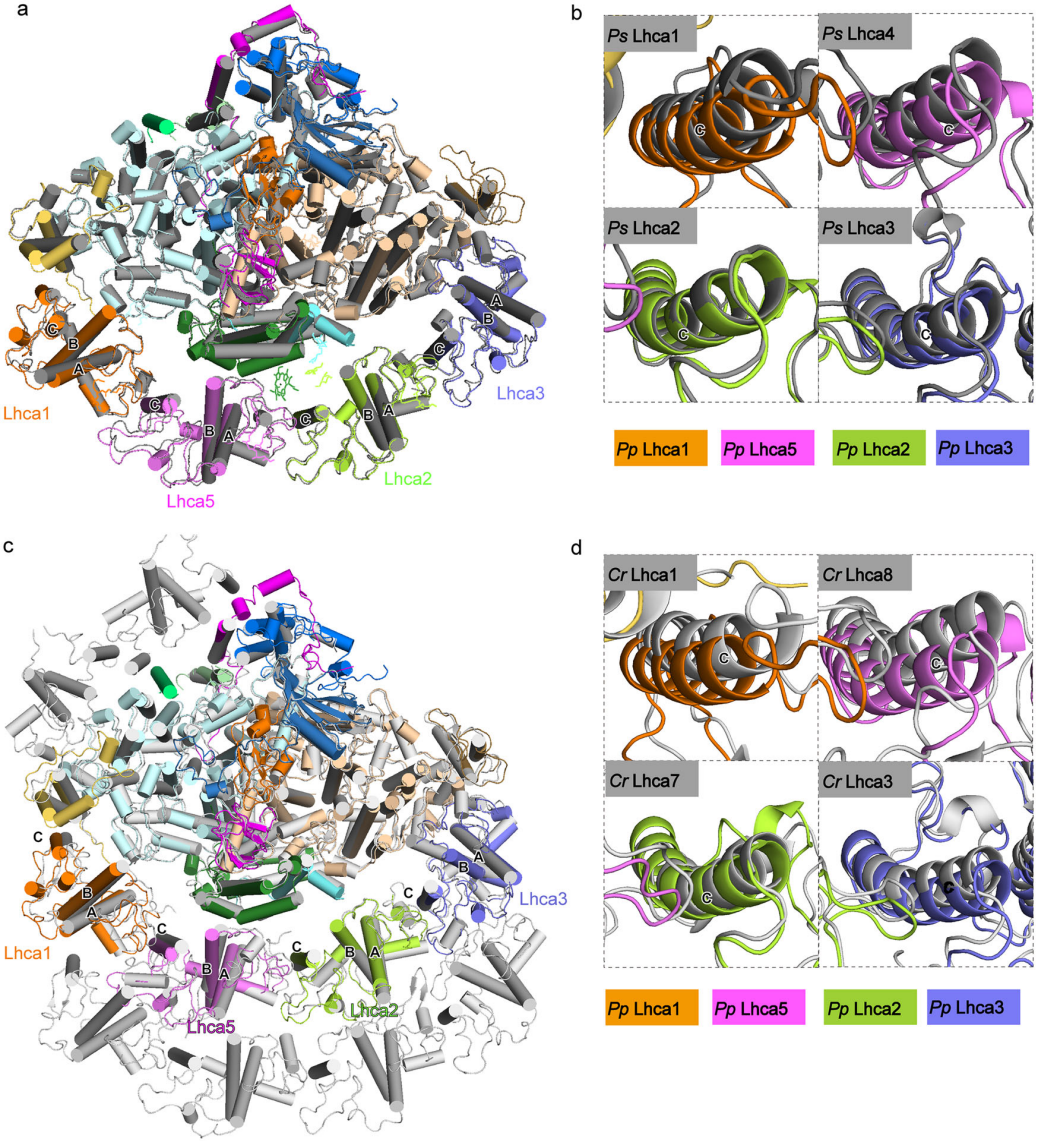
Comparison of the PSI–LHCI structures. **a** Superposition of the PSI–LHCI structures of *P*. *patens* and *P*. *sativum* based on the helices of PsaA/B. Regions of the helix C of each Lhca subunit in panel **a** was enlarged and shown in panel **b**. **c** Superposition of the PSI–LHCI structures of *P*. *patens* and a green algae *C*. *reinhardtii* based on the helices of PsaA/B. Regions of the helix C of each Lhca subunit in panel **c** was enlarged and shown in panel **d**. Color codes and PDB ID codes: *Ps* PSI–LHCI, gray, 4XK8; *Cr* PSI–LHCI, light gray, 6IJO; *Pp* PSI–LHCI, the same color codes as in Fig. 1, 6L35.

Superposition of *Pp* PSI–LHCI and *Ps* PSI–LHCI based on PsaA and PsaB, the two biggest subunits of the PSI core complex, shows a shift in their LHCI belts, with *Pp* LHCI moving away from the core. Among the four *Pp* Lhcas, Lhca5 shows the largest shift, followed by Lhca1, whereas Lhca2 and Lhca3 are hardly shifted (Fig. 6a, b). The shift of LHCI may be caused by *Pp* Lhca5, the absence of PsaN, lipids in the gap region, and some other unknown reasons. In addition, PsaF and PsaG were also shifted slightly away from the core, which may also result in the large shift of Lhca1–Lhca5 (Fig. 6). Green algal PSI–LHCI supercomplex contains one inner LHCI belt surrounding the core and one outer LHCI belt surrounding the inner one^9–11^. Similarly, a shift of *Pp* LHCI relative to the inner LHCI belt of green algae was revealed, when *Pp* PSI–LHCI was compared to the green algal PSI–LHCI (Fig. 6c, d). These results indicate that the gap between LHCI and the core in *P*. *paten* is wider than that in other green plants, which could affect the efficiency of EET from LHCI, especially from the Lhca1–Lhca5 dimer, to the PSI core complex.

### Possible excitation energy-transfer pathways from LHCI to PSI core

In order to clarify features of the pigment network of LHCI and possible EET pathways from LHCI to the PSI core in *P*. *patens*, we compared the distribution of Chls located at LHCI and at the gap region between the LHCI belt and the core among *P*. *patens*, *C*. *reinhardtii*, and *P*. *sativum* (Fig. 4). Most of the Chls in *Pp* LHCI have counterparts in LHCI from the other two organisms, however, Chls of *Pp* LHCI are slightly farer away to the core, as the *Pp* LHCI, especially *Pp* Lhca5, is shifted away from the PsaA/B (Fig. 6).

At the stromal layer, both *P*. *patens* and *P*. *sativum* have two linker Chls *b* located at the interfaces of Lhca1–Lhca5/Lhca4 and Lhca5/Lhca4–Lhca2, and they are Chl *b*615/Lhca5 and Chl *b*615/Lhca2 in *P*. *patens* and Chl *b*615/Lhca4 and Chl *b*614/Lhca2 in *P*. *sativum*. These Chls *b* are absent in the *C*. *reinhardtii* PSI–LHCI, and could mediate energy transfer between different Lhca monomers. In addition, Chl *a*614 of Lhca4 is found only in *P*. *sativum* and is located in the interface between Lhca1 and Lhca4, suggesting that it could mediate energy transfer between Lhca1 and Lhca4 and be important for land plants to adapt to their light conditions.

At the lumenal layer, the Chl distribution in *P*. *patens* is more like *P*. *sativum* than *C*. *reinhardtii*. Two Chls 616 located at the gap region between LHCI and the core in the two middle Lhca subunits Lhca7 and Lhca8 of *C*. *reinhardtii*, are lost in the corresponding Lhca subunits of *P*. *patens* and *P*. *sativum*, and Chl 616 in Lhca1 of the green alga is also somewhat shifted. Green algal PSI–LHCI supercomplex has a large antenna cross-section consisting of 8 or 10 Lhca subunits, and nearly all of them bind Chl 616^9–11^. Considering that red algal Lhca subunits have a similar Chl binding site of Chl 616^6^, it was speculated that loss of the gap Chls 616 may be a strategy for adaption to the terrestrial environment. In spite of the difference in the distribution of Chl 616, one similarity between *P*. *patens* and the green alga is that Lhca3 binds a Chl molecule (Chl 615) in both species, which was not found in *P*. *sativum*.

Chls 603 and 609 are assigned as red-form dimers responsible for the red-shifted fluorescence emission, as those in green algal and other land plant PSI–LHCI. Although the positions of Chls 603 and 609 are conserved, the ligand to Chl 603 could be either His or Asn, which may be responsible for the extent of the fluorescence red-shift, as the ligand Asn leads to a more red-shifted fluorescence emission than His by providing the right geometry between the dimers to allow for their stronger interaction^1^. In *P*. *sativum*, Chl 603 is coordinated by Asn in Lhca3 and Lhca4 and by His in Lhca1 and Lhca2^12^; in green algae, Chl 603 is coordinated by His in each of the four Lhca positions^9–11^, whereas in *P*. *patens*, Chl 603 is coordinated by His_93_, His_117_, and His_110_ in Lhca1, Lhca5, and Lhca2, respectively, and by Asn_148_ in Lhca3. Correspondingly, *P*. *patens* has a fluorescence emission peak that is red-shifted compared to the green alga *B. corticulans* but blue-shifted compared to the plant *P*. *sativum* (Supplementary Fig. S1). These results support the suggestion that the ligand Asn for Chl 603 in Lhca4 is responsible for the most red-shifted spectral forms in land plants^27^.

**Fig. 7:**
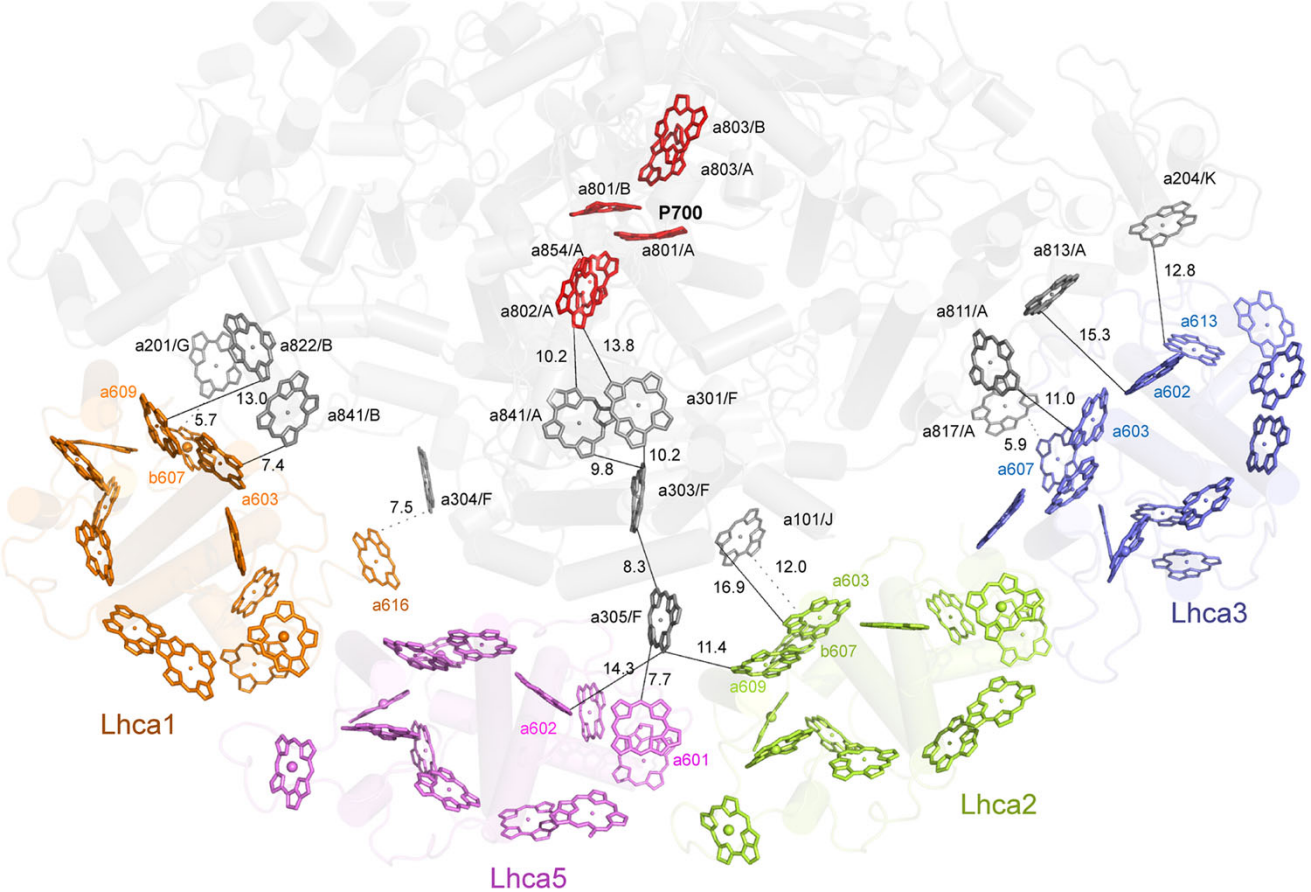
Plausible energy-transfer pathways from LHCI to the PSI core of *P*. *patens*. The color codes for the Chls of each Lhca subunit are the same as Fig. 1. In the PSI core, Chls of the electron transfer chain and reaction center are shown in red, and other Chls are shown in gray. Chls at the stromal layer and lumenal layer are shown in sticks and lines, respectively. Edge-to-edge distances between Chls located at the stromal layer and lumenal layer are indicated by black lines and black dotted lines, respectively. The central magnesium atoms of Chl *b* are shown in large spheres to distinguish them from Chl *a*.

Based on the edge-to-edge distances, main EET pathways from each Lhca to the core complex can be deduced (Fig. 7). In Lhca1, short edge-to-edge distances were found between Chl 603–Chl 841/PsaB (7.4 Å) and Chl 609–Chl 822/PsaB (13 Å) at the stromal side, and Chl 607–Chl 201/PsaG (5.7 Å), Chl 616–Chl 304/PsaF (7.5 Å) at the lumenal side. In the two middle Lhca subunits, Chl 601/Lhca5 and Chl 609/Lhca2 have short edge-to-edge distances with Chl 305/PsaF of 7.7 and 11.4 Å, respectively. Chl 305/PsaF is in an important position because it fills in the large distances between the two middle Lhca subunits with the core, from which excitation energy can be transferred along the pathway consisting of Chl 303/PsaF, Chl 841/PsaA, Chl 301/PsaF, and Chl 802/PsaA; and all edge-to-edge distances between adjoining Chl molecules within this pathway are no longer than 15 Å. By this pathway the harvested light energy can be transferred from Chls in LHCI to Chls in the electron transfer chain. In addition, Chl 603 and Chl 607 of Lhca2 have short edge–edge distances with Chl 101/PsaJ of 12.0 and 16.9 Å at the lumenal and stromal sides, respectively, similar to the pathway in *P*. *sativum*. In Lhca3, the following Chl pairs may provide EET pathway to the core complex: Chl 603–Chl 811/PsaA (11.0 Å) and Chl 602–Chl 813/PsaA (15.3 Å) at the stromal side, and Chl 607–Chl 817/PsaA (5.9 Å) and Chl 613–Chl 204/PsaK (12.8 Å) at the lumenal side. Taken together, the EET pathways from the two-side Lhca subunits in *P*. *patens* are very similar to those seen in *P. sativum*, whereas EET pathways from the two middle Lhca subunits of *P*. *patens* may be more efficient than those in *P*. *sativum*.

## Discussion

The number of Lhca subunits bound to the PSI core of the *Pp* PSI–LHCI supercomplex is the same as those of land plants and also similar to that of a minimum PSI–LHCI found in a green alga^28^, but significantly <8–10, the number of Lhcas found in most green algae^9–11,25^. In most of the green algae, two semi-belts of Lhcas, each consisting of four Lhcas, are associated with the PSI core. The outer Lhca belt was dissociated from PSI after landing during the evolution of green plants. This suggests that reducing the antenna size to half is a result of adaptation from aquatic to terrestrial environment, during which the light intensity is changed from low in the water to high in the air. Therefore, decreasing the capacity of light harvesting under excess light illumination may be a result of protecting land plants from photodamage.

Compared with the Lhca1–Lhca4–Lhca2–Lhca3 arrangement of the Lhca belt in land plants *P*. *sativum* and *Z*. *mays*, the Lhca of *P*. *patens* is arranged in the order of Lhca1–Lhca5–Lhca2–Lhca3. The difference happens in the second Lhca position, namely, Lhca5 in *P*. *patens*, which is replaced by Lhca4 in the land plants. This is consistent with a previous report that although Lhca1, Lhca2, and Lhca3 specifically bind to the PSI core to form the PSI–LHCI supercomplex, only Lhca4 can be partially substituted by Lhca5 in *A*. *thaliana*^29^. The abundance of Lhca5 was also found to increase in *A*. *thaliana* lacking Lhca4^30,31^, and Lhca5 could form a stable dimer with Lhca1 by in vitro reconstitution experiments^32^, which supports a direct interaction of Lhca5 with Lhca1 for binding to the core in the absence of Lhca4. In addition, *Cr* Lhca8 is an *At* Lhca5-like subunit^33^ and binds to the second position of Lhca in *C*. *reinhardtii*, which is occupied by Lhca4 in the land plants, suggesting that the Lhca at this position is changed from Lhca8/Lhca5 in algae and moss to Lhca4 in higher plants.

The energy level of red Chl forms is a critical factor for EET in PSI. For example, comparison between green algal PSI–LHCI and higher plant PSI–LHCI showed that, although the former has significant larger antenna size than the latter in most cases, they have similar average transfer and decay times for excitation energy. This is largely because the energy level of red Chls in green algal LHCI is higher than those in plant LHCI (Supplementary Fig. S1)^34,35^. In land plants, Lhca4 and Lhca3 have fluorescence emission peaks at around 730 nm, and Lhca1 and Lhca2 emit slightly longer wavelength than 700 nm^36–38^. In contrast, Lhca5 emits at 684 nm^32^, which is slightly red-shifted compared to the antenna of PSII (680 nm). Thus, Lhca4 has red forms with the lowest energy level, whereas Lhca5 has the highest energy level. It was inferred that the presence of red Chls can slow down the EET efficiency, and the lower their energy is, the longer it will take to transfer energy from the red forms to bulk Chls^1^. Therefore, the substitution of Lhca5 by Lhca4 in the second Lhca position of PSI–LHCI supercomplex is suggested to slow down the EET from Lhca at this position to the core complex.

In addition to the feature of binding red Chls, Lhca4 lacks an efficient EET pathway to the core complex. However, compared with *Pp* Lhca5, it binds one more Chl molecule (Chl 614), which has been suggested to play a key role in transferring energy from Lhca4 to the core via Lhca1^12^. By contrast, in *P*. *patens*, Chl 305/PsaF provides a bridge to connect Chls of both Lhca5 and Lhca2 with the core complex, facilitating EET from Lhca5 and Lhca2 to the core directly. These structural differences, together with the differences in energy level of red forms, suggest that the light-harvesting and EET process from LHCI to the core is more efficient in *P*. *patens* than in other land plants bearing Lhca4 subunit. Future time-resolved measurements are needed to prove this. The possible physiological function of LHCI in *P*. *patens* may be related to its living environment with a low light and high humidity, where fast EET may be beneficial for its survival. In case of strong light, excess absorbed energy can be dissipated by strong non-photochemical quenching that depends on both PsbS and LHCSR^39,40^. On the contrary, Lhca4 appeared later in the evolution and may have a relatively less efficient EET to the core, which may help land plants to survive under strong light conditions.

The function of Lhca5 has changed largely during evolution, that is from one main component of the Lhca belt in green algae and moss to a minor Lhca involved in cyclic electron flow in higher plants. Although Lhca5 has been shown to bind to the outside of Lhca2/Lhca3 subunits of the PSI complex at the sub-stoichiometric level in *A*. *thaliana* wild type^41^, neither Lhca5 nor Lhca6 is the component of Lhca belt connected with the core directly in higher plants. However, they are required to connect PSI–LHCI with NADH dehydrogenase-like (NDH) complex to form intact PSI–NDH supercomplex, which is essential for the NDH-dependent cyclic electron flow^42^. Functional experiments have suggested that the specific function of connection between PSI and NDH can be fulfilled by either Lhca5 and Lhca6^43^. In *P*. *patens*, there is no gene corresponding to *At-lhca6* and only a small part of NDH are associated with PSI–LHCI^44^. In another model bryophyte, liverwort *Marchantia polymorpha*, there are no genes corresponding to *At-lhca5* and *At-lhca6*, and NDH does not interact directly with PSI^45^, suggesting that the involvement of Lhca5 and Lhca6 in the NDH–PSI supercomplex is a relatively recent evolutionary event. Since cyclic electron flow has been suggested to balance the ratio of ATP/NADPH production and to protect the photosystems from damage under excess illumination or fluctuating light conditions^46,47^, the NDH–PSI supercomplex could be a result of adaptation to the land environment.

Bryophytes are the first photosynthetic organisms appeared in the land, and therefore have important biological significance in the process of plant evolution^16^. Our results reveal that the structure of PSI–LHCI from *P*. *patens* is largely similar to that of higher plants, but also has distinct features that may play important roles in EET under the unique environment that is humid and fluctuating in light intensities. These results may provide clues for understanding the relationship between structure and function of PSI–LHCI during evolution from aquatic algae to the early land plants, and then to the well-adapted land plants.

## Materials and methods

### Sample purification and characterization

*P*. *patens* ecotype Grandsen 2004 was grown on a layer of cellophane overlaid on BCDAT agar medium supplemented with glucose (5 g/L) at 25 °C under continuous light at about 50 µ mol photons m^−2^ s^−1^. Protonemata (14–21 days old) was harvested, flash frozen in liquid nitrogen, and stored at −80 °C for further use. Crude thylakoids were isolated as described previously^16,48^. The crude PSI–LHCI supercomplex was isolated from the thylakoid membranes by a strong anion-exchange column (Q Sepharose High-Performance; GE Healthcare) (Supplementary Fig. S1a), followed by purification with a sucrose density gradient centrifugation (0–1.0 M sucrose solution containing 20 mM Tricine-NaOH, pH 7.8, and 0.03% β-DDM) at 150,000 × *g* for 17 h at 4 °C (Supplementary Fig. S1b). The dark green band (Supplementary Fig. S1b) after sucrose density centrifugation was collected for cryo-EM study.

The polypeptide composition of PSI–LHCI from *P*. *patens* was analyzed by SDS-PAGE. Samples were treated with a sample buffer containing 2% (w/v) lithium dodecyl sulfate, 60 mM dithiothreitol, and 60 mM Tris-HCl (pH 8.5) at 60 °C for 10 min, and subjected to SDS-PAGE with a 16% gel containing 7.5 M urea^49^.

### Sequence alignments and phylogenetic analysis

All the sequences of PSI–LHCI subunits of *P*. *patens* are blasted by NCBI https://www.ncbi.nlm.nih.gov/. Multiple sequence alignment was performed by CLUSTALW and ENDscript 3.0^50^, and the evolutionary analysis of all *lhca* genes from *P*. *patens* and *A*. *thaliana* was performed by phylogeny (http://www.phylogeny.fr/simple_phylogeny.cgi).

### Mass spectrometry (MS) analysis

The single bands of protein subunits were obtained by SDS-PAGE electrophoresis and cut out, subjected to mass analysis using a matrix-assisted laser desorption ionization time-of-flight mass spectrometer MALDI-TOF/TOF (UltraflextremeTM, Brucker, Germany). The protein samples were first treated with DTT reduction and alkylation with iodoacetamide, followed by treatment with trypsin overnight^51^. The peptides obtained after enzymatic hydrolysis were desalted by a C18 ZipTip and mixed with the matrix-cyano-4-hydroxycinnamic acid, followed by mass analysis. Database searches were performed through the MS/MS Ion Search page at www.matrixscience.com. The protein identification results were obtained based on the primary mass spectrometry and the secondary mass spectra of the peptides produced after enzymatic hydrolysis. The database used for the identification is NCBIProt, swissprot.

### Cryo-EM data collection

The concentration of *P*. *patens* PSI–LHCI was set to around 3 mg Chl per ml, and 4 μl of the sample was applied to glow-discharged Quantifoil R1.2/1.3 400-mesh holey carbon film grids (Quantifoil, Micro Tools GmbH) in an FEI Vitrobot Mark IV at 100% humidity and 8 °C under green light. After incubating for 30 s, the grids were blotted for 3 s with blotting force at level 2 and immediately plunge-frozen in liquid ethane. Samples were screened using a FEI Tecnai Arctica TEM 200 kV electron microscope equipped with FEI Falcon II direct electron detector, and 4052 micrographs were acquired using the EPU software (FEI) at a magnification of 78,000, corresponding to a pixel size of 1.27 Å. The defocus range was between 2.0 and 2.5 μm, and micrographs with 1.6 s exposure time had 26 dose-fractioned frames, and the total dose was ~50 e^–^Å^–2^.

For higher resolution structural analysis, the same sample was imaged on an FEI Titan Krios electron microscope operated at 300 kV with a K2 Summit direct electron detector (Gatan) in the super-resolution counting mode. A GIF Quantum energy filter was used and the slit was set to 20 eV. Micrographs were recorded at a magnification of 130,000 with a pixel size of 1.061 Å and their defocus values were varied from 1.5 to 2.5 μm. Micrographs were dose-fractioned into 32 frames with a total exposure time of 8 s, having a total dose of ~50 e^–^Å^–2^. In total, 6952 micrographs were collected using AutoEMationII automated data collection software^52^.

### Data processing

All micrographs were processed for motion correction by MotionCorr2^53^ with dose weighting, and the defocus extents were estimated by CTFFIND4^54^ using the micrographs without dose weighting. All other steps of image processing were performed using RELION 3.0^55^. For the data from FEI Tecnai Arctica TEM, 4052 micrographs were selected and about 1500 particles were manually picked from several micrographs. These particles were subjected to 2D classification, and three selected images representing projections of PSI–LHCI in different orientations were used as templates for automated particle picking. In total, 537,999 particles were auto-picked from the selected micrographs. All extracted particles were binned two times (leading to 2.54 Å/pixel) and subjected to 2D classification, and 13,500 particles were selected to generate an initial model. After 2D classification, a total of 494,918 particles were finally selected for 3D classification. One main class showed good secondary structural features and was selected. An auto-refinement procedure was carried out using the particles from the main class, resulting in a reconstruction at 5.9 Å resolution. These particles were re-extracted into the original pixel size of 1.27 Å. After 3D refinement without any symmetry imposed and particle polishing, the resulting 3D reconstructions from 245,039 particles yielded an EM map at 4.1 Å resolution using the gold-standard Fourier shell correlation (FSC) = 0.143 criteria.

For the data from FEI Titan Krios TEM, 3712 micrographs with the maximum resolution above 5 Å were selected. Using the 2D templates from the data obtained above, 535,146 particles binned two times (leading to 2.122 Å/pixel) were auto-picked from the selected micrographs. The number of particles was reduced to 455,383 by two rounds of 2D classification. The reconstructions with 4.1 Å resolution obtained above were low-pass filtered to 60 Å and then was used as the references of the 3D classifications. One main class (49.5%) was selected and auto-refined without any symmetry imposed, which resulted in a reconstruction at an overall resolution of 9.7 Å. Then its 225,998 particles were re-centered and subjected to 2D classification, from which 202,246 particles were selected for further 3D classification using the 9.7 Å reconstruction low-pass filtered to 60 Å as the 3D reference. Four of the 3D classes showed good secondary structural features, and were selected and their 121,326 particles were combined and re-extracted into the original pixel size of 1.061 Å. After 2D classification, 116,247 particles were selected and auto-refined without any symmetry imposed to reconstruct a map at an overall resolution of 4.6 Å. Then this map was used as the reference without low-pass filter for further 3D classification, and the main class with 70,288 particles was auto-refined to yield a 4.38 Å reconstruction. After contrast transfer function (CTF) refinement and post-processing, a 3.23 Å cryo-EM map with the gold-standard Fourier shell correlation (FSC) = 0.143 criteria was gained.

### Model building, refinement, and validation

The 3.23 Å cryo-EM map of *P*. *patens* PSI–LHCI complex was used for model building; it was of good quality for de novo atomic model building. Since the structure of the pea PSI core taken from the PDB 4XK8^12^ fits nicely with the cryo-EM map using UCSF Chimera^56^, it was used as a guide to build the model. The models of LHC proteins were predicted using the Phyre2 web server (http://www.sbg.bio.ic.ac.uk/phyre2) and were then fitted into the cryo-EM map. The overall model of PSI–LHCI was refined against summed maps using phenix real space refine with secondary structure restraints applied^57^, and was checked and corrected manually again with COOT^58^. The resulting model was validated to avoid overfitting using the methods as described previously^59^.

## Supplementary information

Fig S1

Fig S2

Fig S3

Fig S5

Fig S4

Fig S6

Fig S7

Fig S8

Fig S9

Fig S10

Fig S11

Fig S12

Fig S13

Table S1

Table S2

Table S3

## Supplementary information

**Supplementary Information** accompanies the paper at (10.1038/s41421-021-00242-9).
