# Supplementary material for: Antenna arrangement and energy-transfer pathways of PSI–LHCI from the moss Physcomitrella patens
Source: Cell Discov. 2021 Feb 16;7:10. doi: 10.1038/s41421-021-00242-9 (PMC7884438; doi:10.1038/s41421-021-00242-9)
Supplement: Supplementary file 1 — Fig S1 [file 41421_2021_242_MOESM1_ESM.pdf]

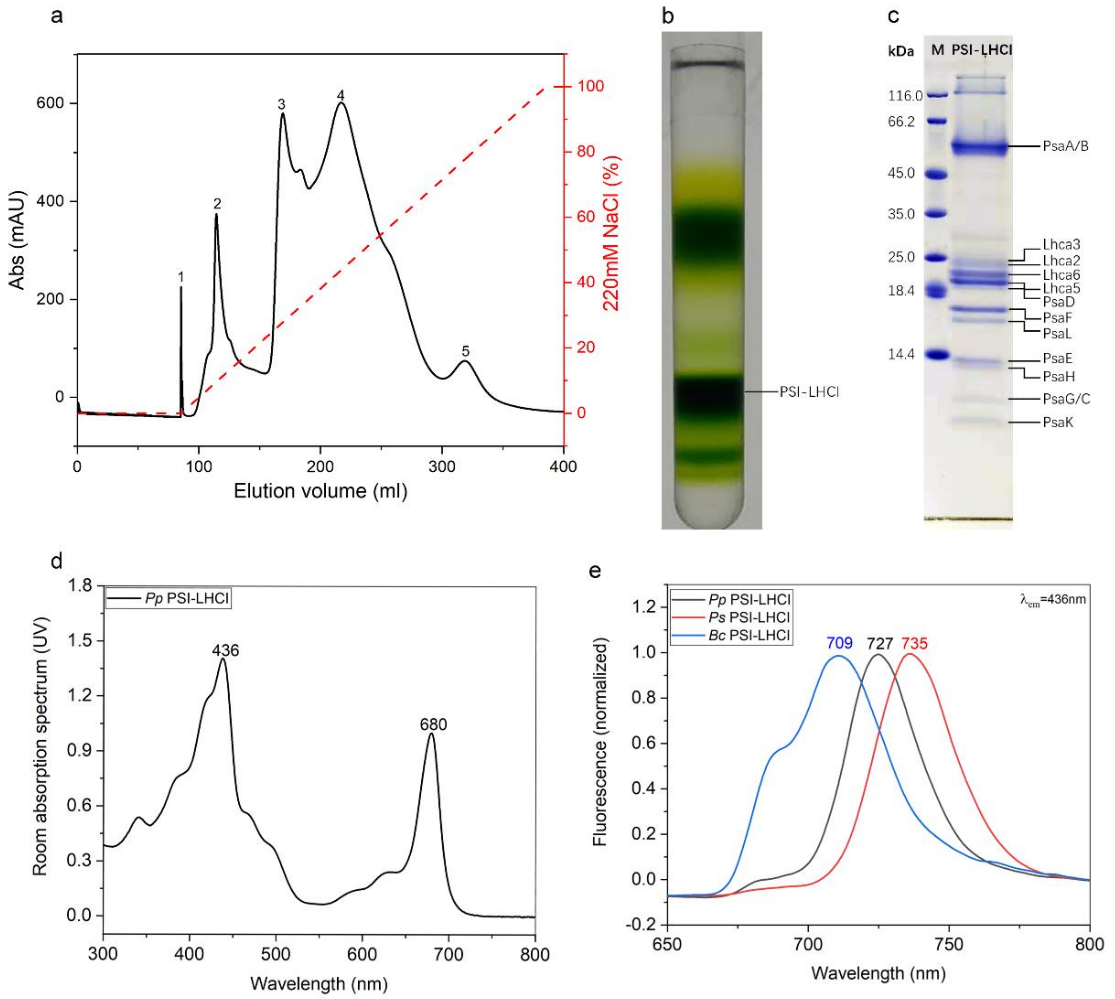

Supplementary Fig. S1 Isolation and characterization of PSI-LHCI super-complex from *P. patens.* **a** Elution pattern of the crude PSI-LHCI from the Q Sepharose High Performance anion-exchange column. The dark green peak 4 mainly contained PSI-LHCI and was collected for further purification. **b** Purification of PSI-LHCI by sucrose density gradient centrifugation. The band labeled PSI-LHCI was pooled and analyzed further. **c** SDS-PAGE analysis of the PSI-LHCI sample prepared from *P. patens*. All bands resolved in the gel were analyzed by mass spectrometry and labelled accordingly. The amino acid sequences of *Pp* Lhca6 show a higher similarity to *At* Lhca1 than to *At* Lhca6 (Supplementary Figs. S5 and S6a), and in fact it was named as Lhca1 according to the genome information of *P. patens* v3.3 in Phytozome. Therefore we used Lhca1 instead of Lhca6 in building the structure. **d** Room temperature absorption spectrum of *Pp* PSI-LHCI. **e** Low-temperature (77 K) fluorescence emission spectra of PSI-LHCI from moss *P. patens* (black), angiosperm *P. sativum* (red), a green alga *Bryopsis corticulans* (blue). These experiments were performed for more than ten times, and the same results were obtained reproducibly.
