# Supplementary material for: Antenna arrangement and energy-transfer pathways of PSI–LHCI from the moss Physcomitrella patens
Source: Cell Discov. 2021 Feb 16;7:10. doi: 10.1038/s41421-021-00242-9 (PMC7884438; doi:10.1038/s41421-021-00242-9)
Supplement: Supplementary file 2 — Fig S2 [file 41421_2021_242_MOESM2_ESM.pdf]

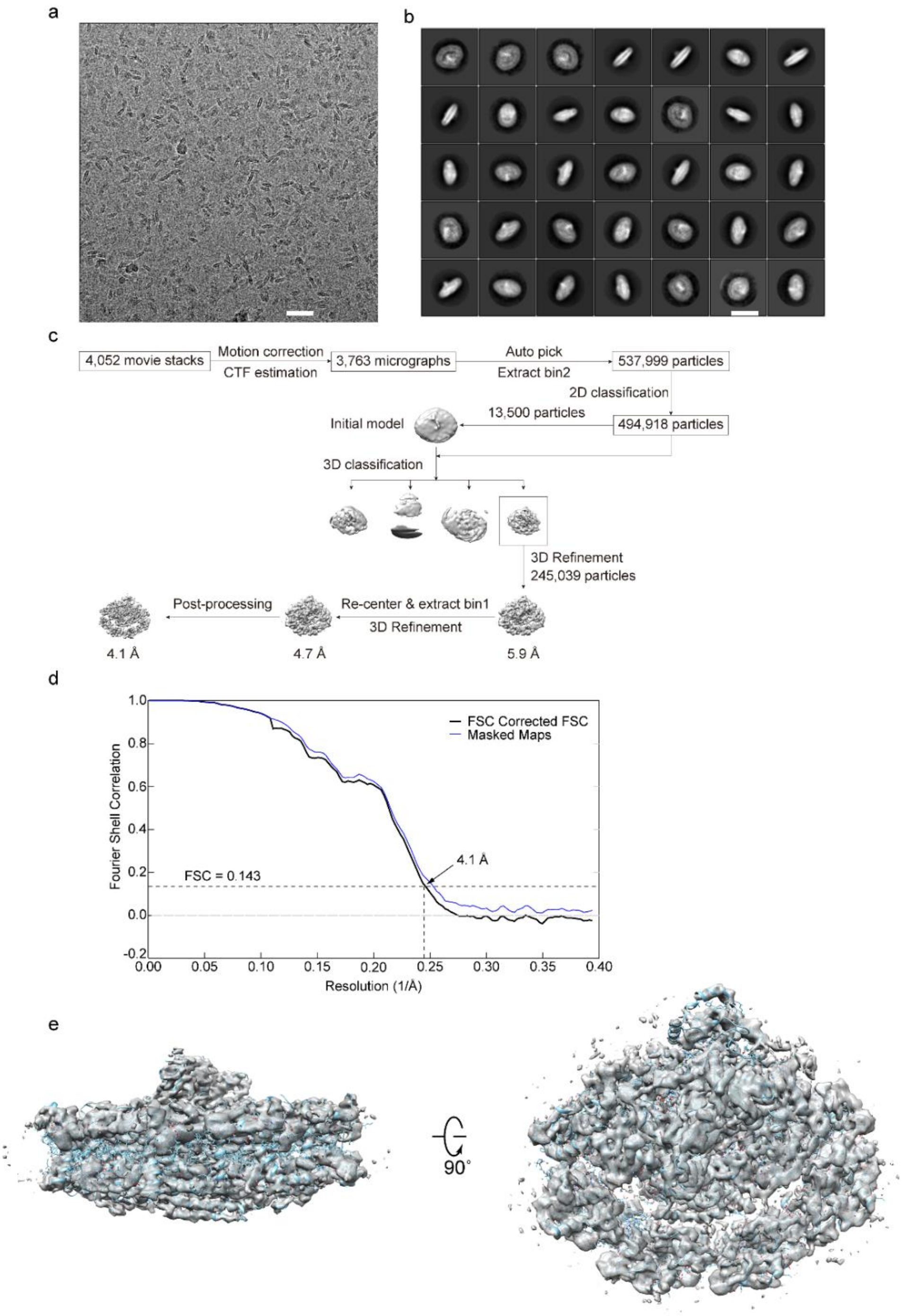

Supplementary Fig. S2 Cryo-EM analysis of *Pp* PSI-LHCI using Tecnai Arctica microscope and FEI Falcon II direct electron detector. a A representative motion-corrected electron micrograph of the *Pp* PSI-LHCI supercomplex. Scale bar, 50 nm. **b** Typical good, reference-free 2D class averages from single-particle images of *Pp* PSI-LHCI. Scale bar, 20 nm. **c** Flowchart of data processing to obtain the 4.1 Å resolution map of *Pp* PSI-LHCI. Details can be seen in the "Data processing" section of MATERIALS AND METHODS. **d** Gold standard FSC curve of the final cryo-EM map with a value of 0.143 at a resolution of 4.1 Å. **e** The crystal structure of *Ps* PSI-LHCI (PDB ID code 4XK8) can be fitted well with the density map of *Pp* PSI-LHCI in different views.
