# Supplementary material for: Antenna arrangement and energy-transfer pathways of PSI–LHCI from the moss Physcomitrella patens
Source: Cell Discov. 2021 Feb 16;7:10. doi: 10.1038/s41421-021-00242-9 (PMC7884438; doi:10.1038/s41421-021-00242-9)
Supplement: Supplementary file 3 — Fig S3 [file 41421_2021_242_MOESM3_ESM.pdf]

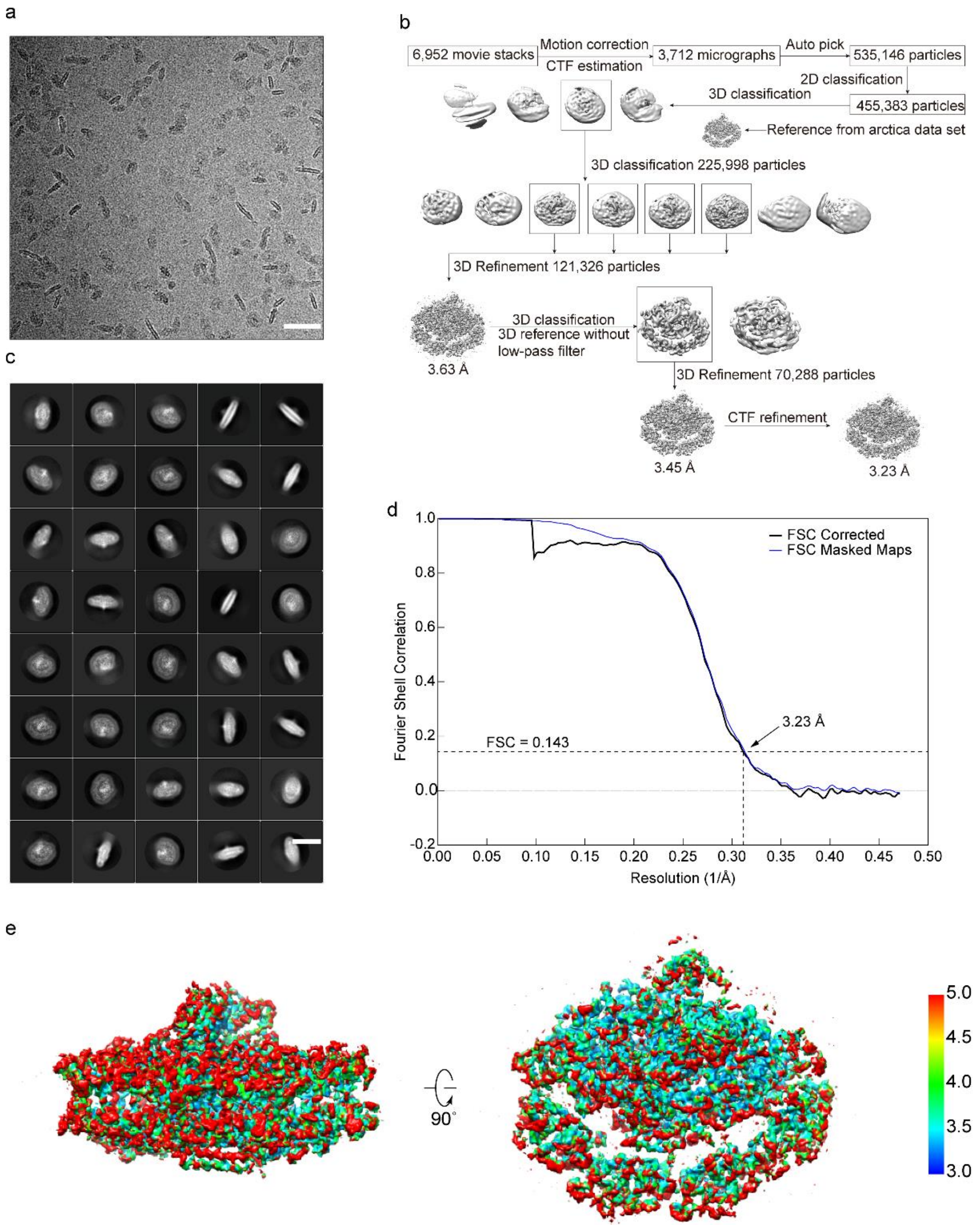

Supplementary Fig. S3 Cryo-EM analysis of *Pp* PSI-LHCI supercomplex using the Tecnai Krios microscope and Gatan K2 Summit direct electron detector. a A representative motion-corrected cryo-EM micrograph of the *Pp* PSI-LHCI supercomplex. Scale bar, 50 nm. **b** Flowchart of data processing to obtain the 3.23 Å resolution map of the *Pp* PSI-LHCI supercomplex. Details can be seen in the "Data processing" section of the MATERIALS AND METHODS. **c** Typical good, reference-free 2D class averages from single particle *Pp* PSI-LHCI images. Scale bar, 20 nm. **d** Gold standard FSC curve of the final cryo-EM map with a value of 0.143 at a resolution of 3.23 Å. **e** Local resolution in side and top views of the *Pp* PSI-LHCI supercomplex estimated with ResMap.
