# Supplementary material for: Antenna arrangement and energy-transfer pathways of PSI–LHCI from the moss Physcomitrella patens
Source: Cell Discov. 2021 Feb 16;7:10. doi: 10.1038/s41421-021-00242-9 (PMC7884438; doi:10.1038/s41421-021-00242-9)
Supplement: Supplementary file 4 — Fig S5 [file 41421_2021_242_MOESM4_ESM.pdf]

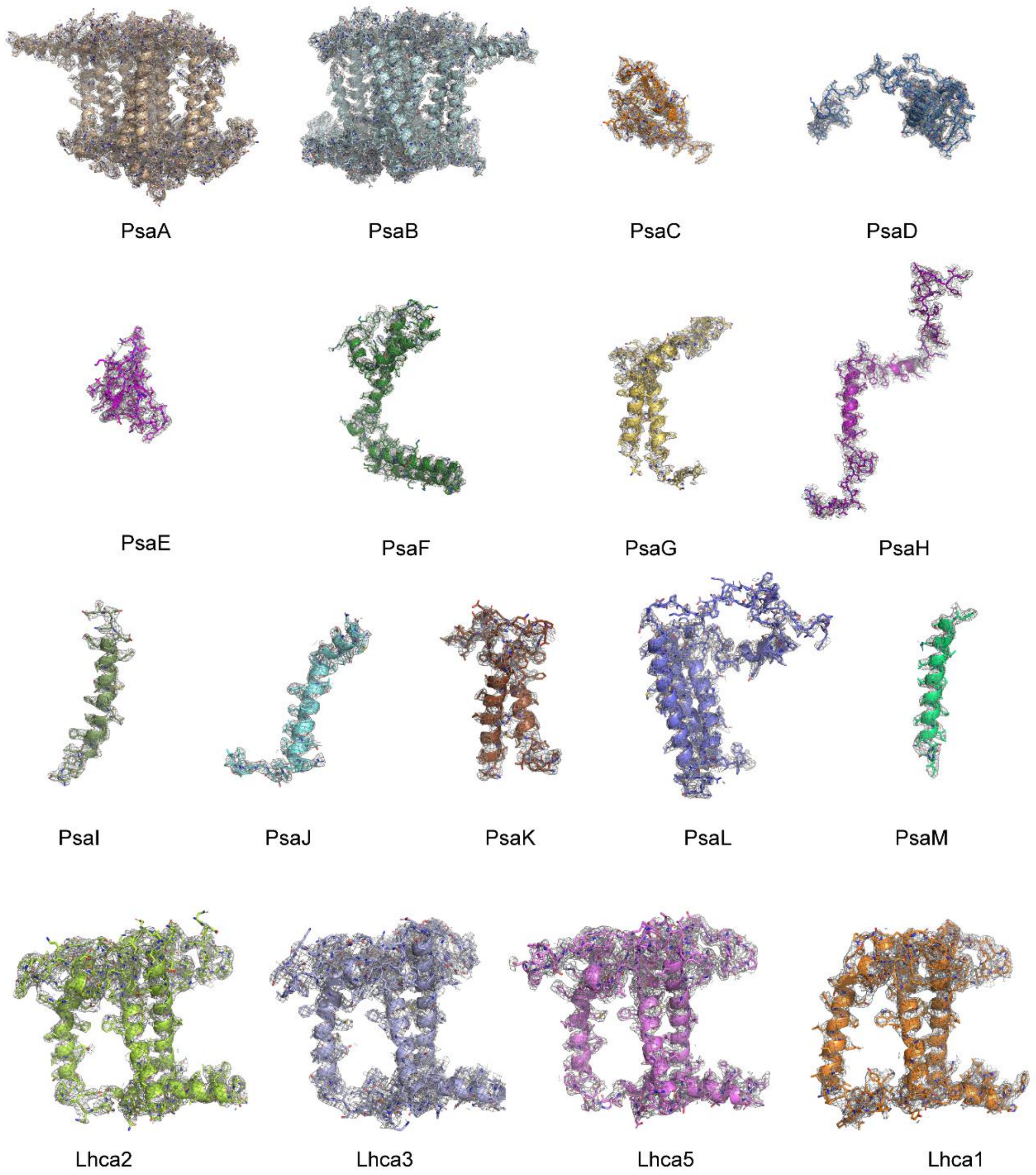

Supplementary Fig. S4 Cryo-EM densities and structural models of the PSI core subunits and Lhca subunits. The subunits are shown as cartoon and colored the same as in Fig. 1. The cryo-EM density map of each subunit is depicted in grey meshes.
