# Supplementary material for: Antenna arrangement and energy-transfer pathways of PSI–LHCI from the moss Physcomitrella patens
Source: Cell Discov. 2021 Feb 16;7:10. doi: 10.1038/s41421-021-00242-9 (PMC7884438; doi:10.1038/s41421-021-00242-9)
Supplement: Supplementary file 5 — Fig S4 [file 41421_2021_242_MOESM5_ESM.pdf]

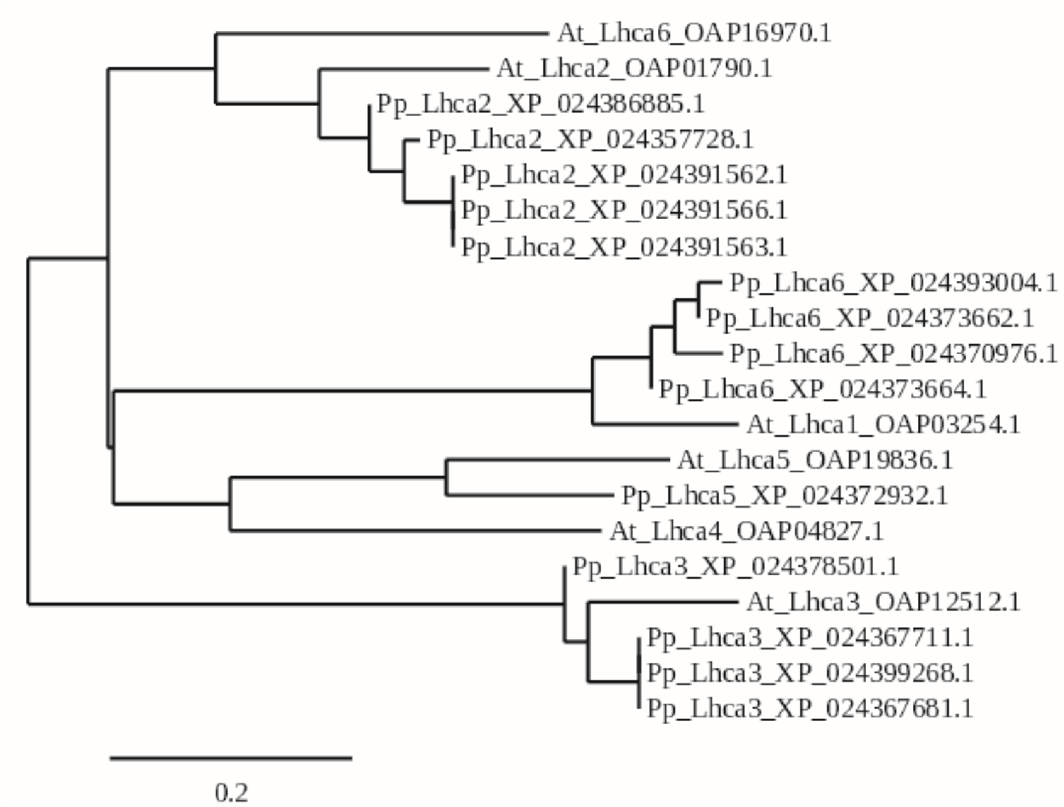

Supplementary Fig. S5 Phylogenetic tree of Lhca proteins. The analysis includes all identified Lhca proteins found in *A. thaliana* (*At*) and *P. patens* (*Pp*). The amino acid sequence of *Pp* Lhca6 was named as Lhca1 according to the genome information of *P. patens* v3.3 in Phytozome and shows a higher similarity to *At* Lhca1 than to *At* Lhca6.
