# Supplementary material for: Antenna arrangement and energy-transfer pathways of PSI–LHCI from the moss Physcomitrella patens
Source: Cell Discov. 2021 Feb 16;7:10. doi: 10.1038/s41421-021-00242-9 (PMC7884438; doi:10.1038/s41421-021-00242-9)
Supplement: Supplementary file 7 — Fig S7 [file 41421_2021_242_MOESM7_ESM.pdf]

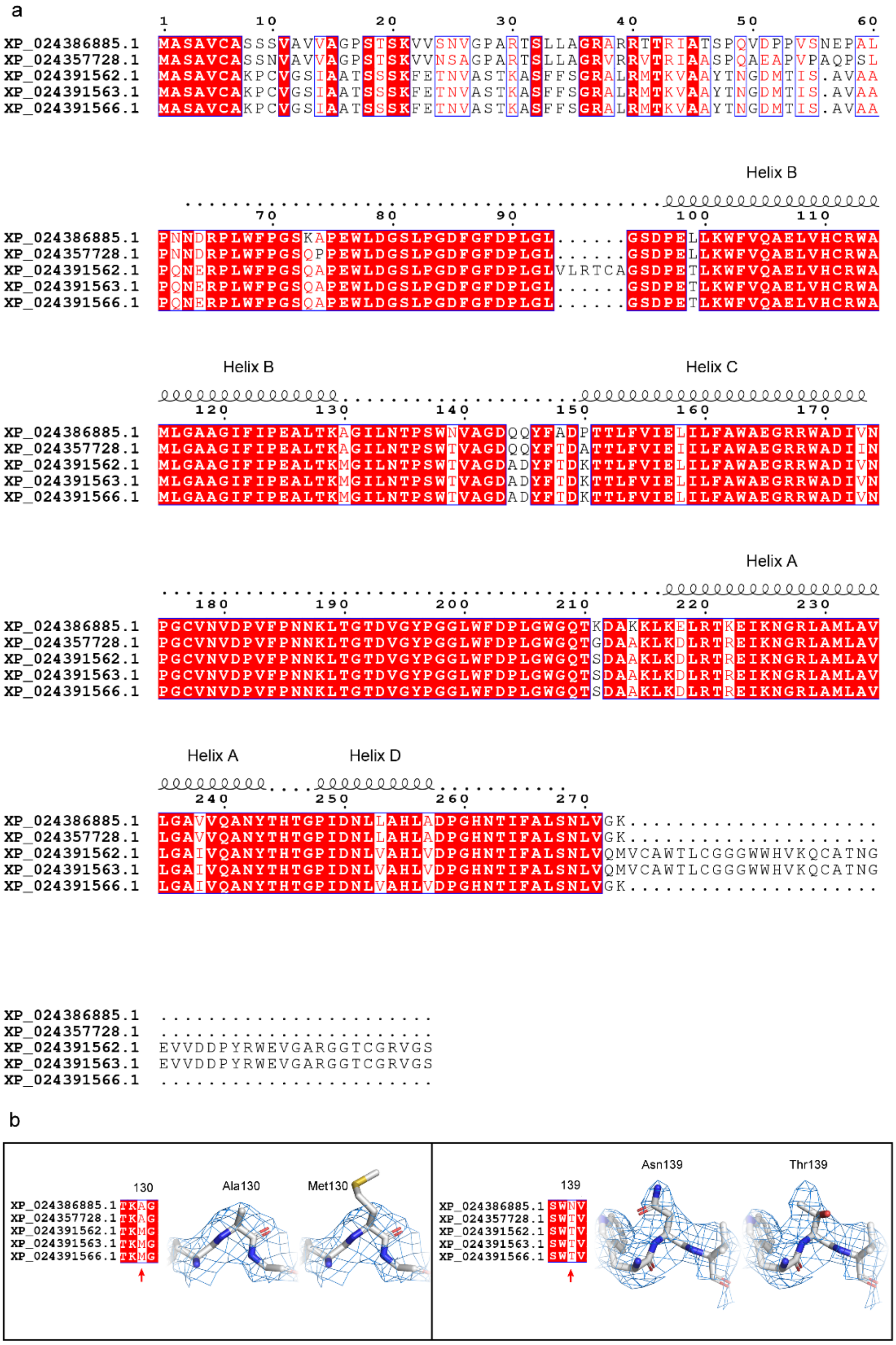

Supplementary Fig. S7 Sequence alignment of *Pp* Lhca2 isoforms and the assignment of the protein subunit in the cryo-EM structure of PSI-LHCI from *P. patens*. **a** Sequence alignment of *Pp* Lhca2 isoforms. **b** Selection of the matched sequence (xp_024386885.1) based on the electron density at variable amino acid residues.
