# Supplementary figures and images for: Antenna arrangement and energy-transfer pathways of PSI–LHCI from the moss Physcomitrella patens
Source: Cell Discov. 2021 Feb 16;7:10. doi: 10.1038/s41421-021-00242-9 (PMC7884438; doi:10.1038/s41421-021-00242-9)

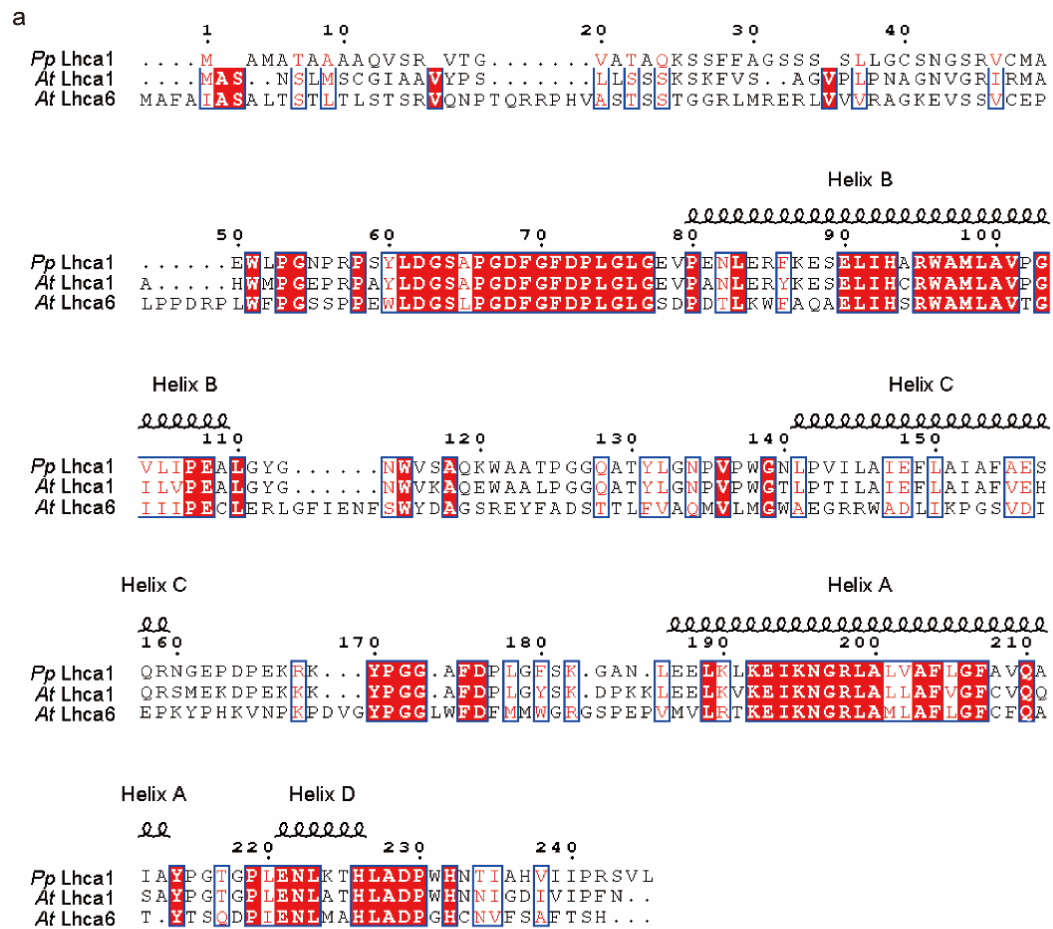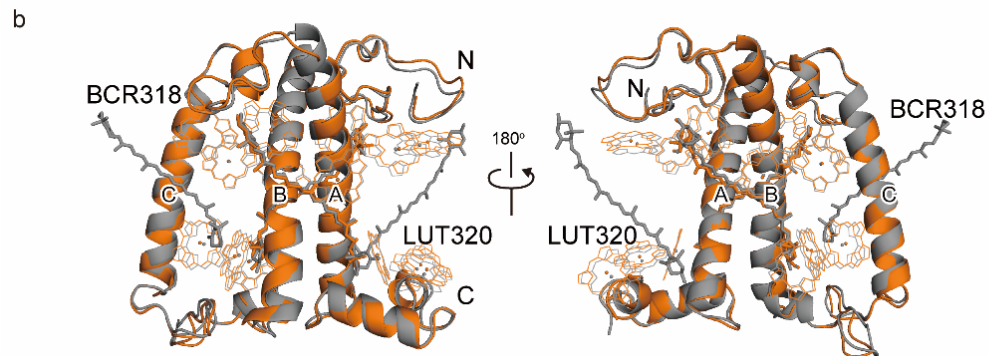

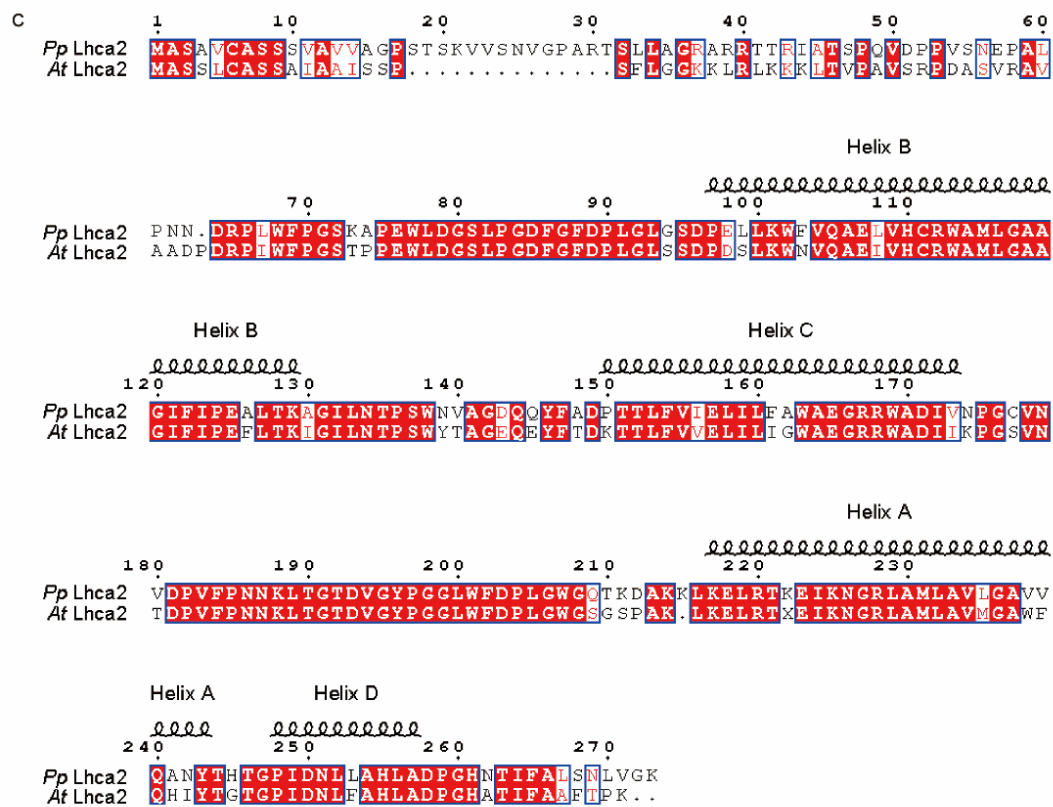

d

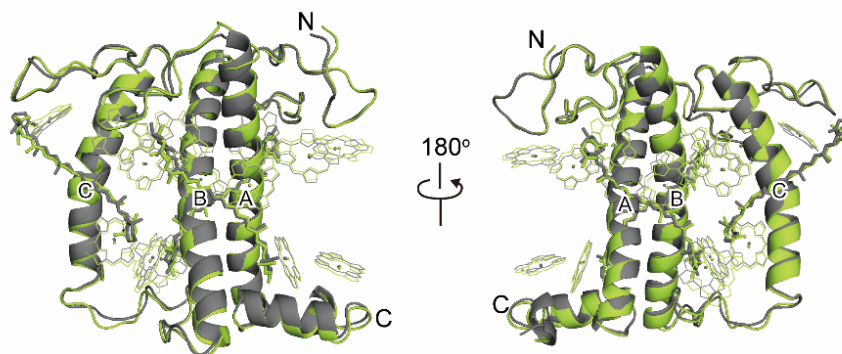

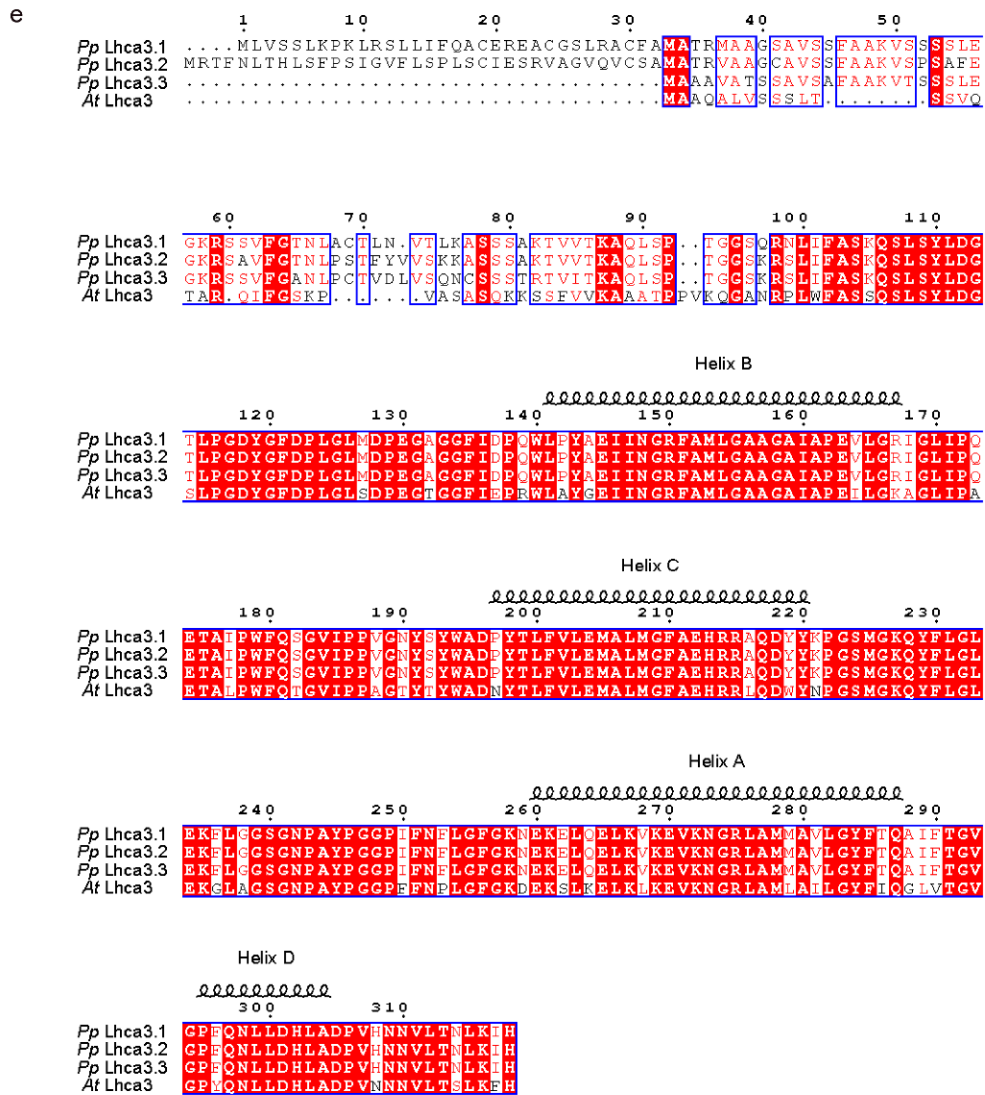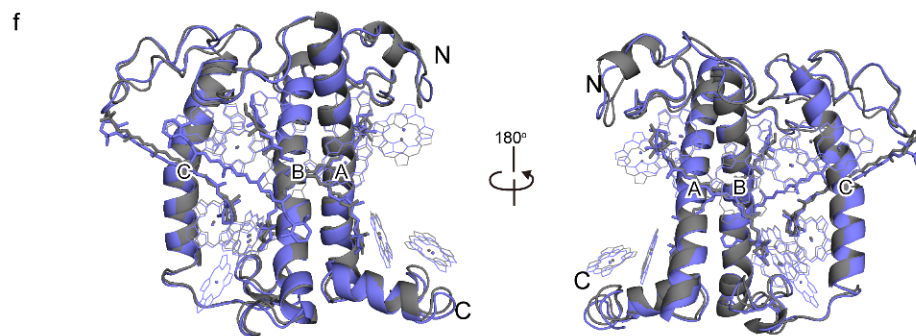

Supplement: Supplementary file 9 — Fig S9 [file 41421_2021_242_MOESM9_ESM.pdf]
