# Supplementary material for: Antenna arrangement and energy-transfer pathways of PSI–LHCI from the moss Physcomitrella patens
Source: Cell Discov. 2021 Feb 16;7:10. doi: 10.1038/s41421-021-00242-9 (PMC7884438; doi:10.1038/s41421-021-00242-9)
Supplement: Supplementary file 10 — Fig S10 [file 41421_2021_242_MOESM10_ESM.pdf]

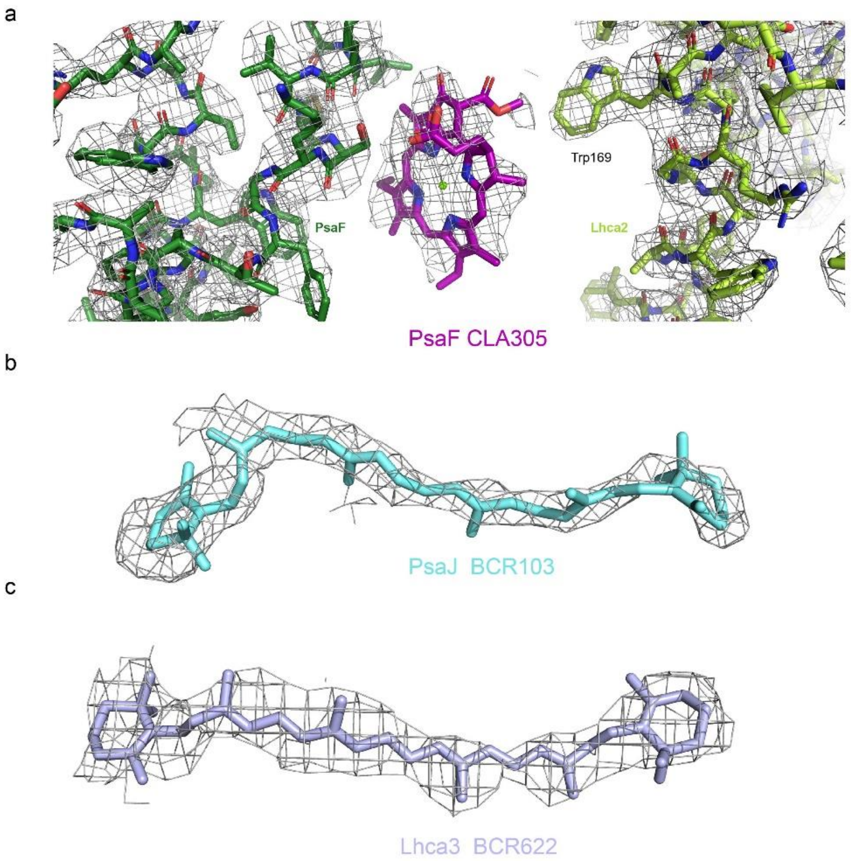

Supplementary Fig. S10 Cryo-EM densities of some newly identified pigments in the present study. **a**, **b** and **c** The cryo-EM density (contour level 0.03) of the newly identified Chl molecule in PsaF (**a**) and two β-carotene molecules in PsaJ (**b**) and Lhca3 (**c**).
