# Supplementary material for: Antenna arrangement and energy-transfer pathways of PSI–LHCI from the moss Physcomitrella patens
Source: Cell Discov. 2021 Feb 16;7:10. doi: 10.1038/s41421-021-00242-9 (PMC7884438; doi:10.1038/s41421-021-00242-9)
Supplement: Supplementary file 11 — Fig S11 [file 41421_2021_242_MOESM11_ESM.pdf]

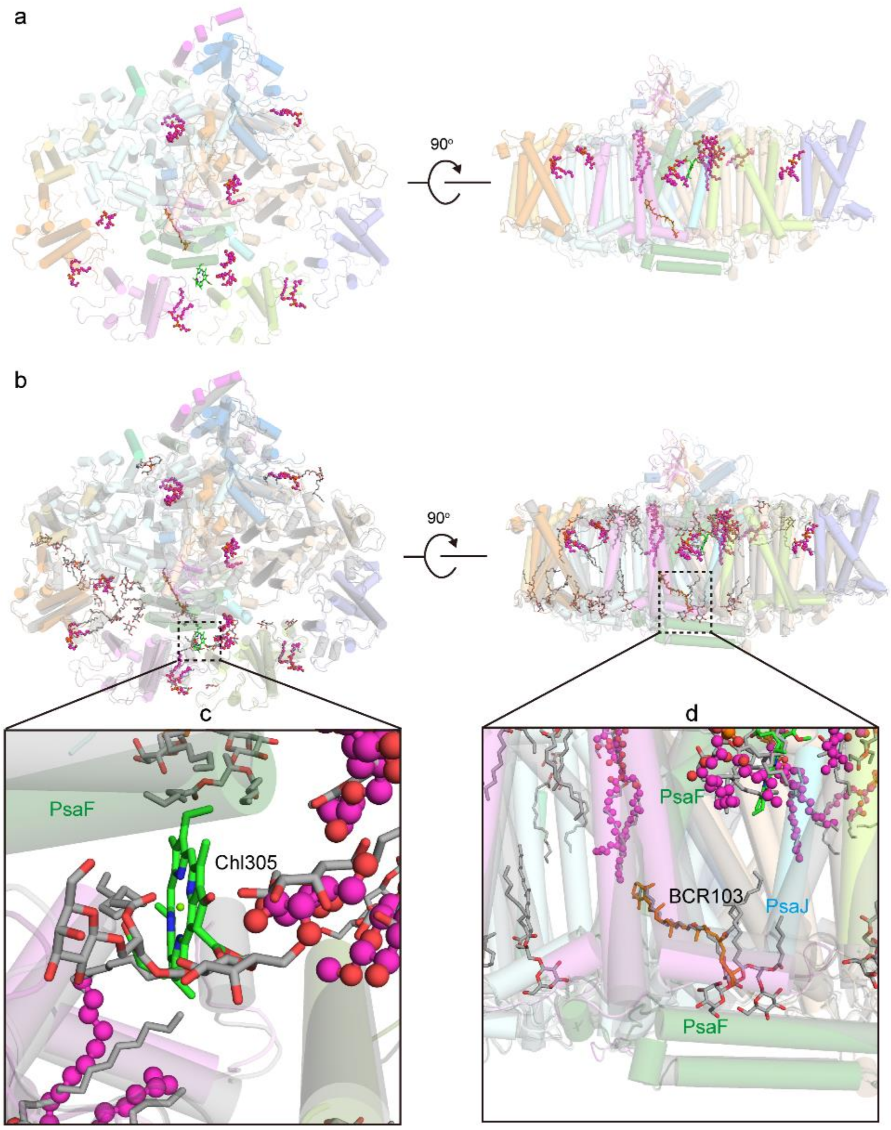

Supplementary Fig. S11 The distribution of membrane lipids in *Pp* PSI-LHCI. a The distribution of membrane lipids LHG, LMG and DGD in *Pp* PSI-LHCI. **b** Comparison of the distribution of membrane lipids between *Pp* PSI-LHCI and *Ps* PSI-LHCI. Regions including the distinctive pigments Chl *a*305/PsaF and BCR103/PsaJ in panel **b** were enlarged and shown in panel **c** and **d** respectively. Color codes and PDB ID codes: *Pp* PSI-LHCI, color is the same as Fig.1, 6L35; *Ps* PSI-LHCI, grey, 5L8R. Carbon atoms of lipids in *Pp* PSI-LHCI and *Ps* PSI-LHCI are depicted in purple sphere and grey sticks, respectively. Oxygen atoms in all lipids are depicted in red.
