# Supplementary material for: Antenna arrangement and energy-transfer pathways of PSI–LHCI from the moss Physcomitrella patens
Source: Cell Discov. 2021 Feb 16;7:10. doi: 10.1038/s41421-021-00242-9 (PMC7884438; doi:10.1038/s41421-021-00242-9)
Supplement: Supplementary file 12 — Fig S12 [file 41421_2021_242_MOESM12_ESM.pdf]

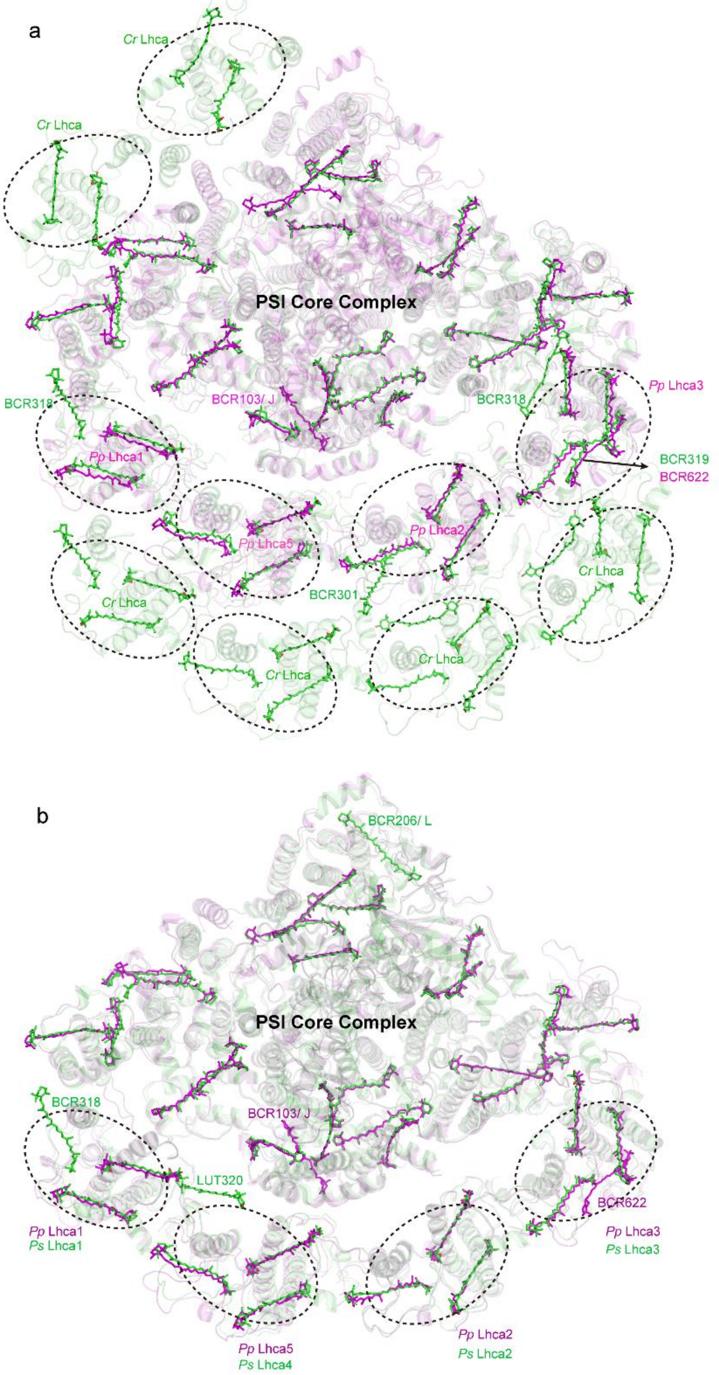

Supplementary Fig. S12 Comparison of pigment arrangements of different PSI-LHCI structures. a Structural comparison of PSI-LHCI from the green alga (*C. reinhardtii*) and *P. patens.* The four Lhca proteins constituting the inner antenna ring of *Cr* PSI-LHCI and the four Lhca proteins of *Pp* PSI-LHCI are nearly overlapped, and they are circled and labelled as *Pp* Lhcas, and other Lhca proteins are circled and labelled as *Cr* Lhcas. Carotenoids bound at different positions are labeled, and one conserved carotenoid binding site (BCR319 in *Cr* PSI-LHCI and BCR622 in *Pp* PSI-LHCI) is also labeled. Color codes and PDB ID codes: *Cr* PSI-LHCI, green, 6IJO; *Pp*

PSI-LHCI, purple, 6L35. **b** Structural comparison of PSI-LHCI from *P. sativum* and *P. patens*. *Pp* Lhcas and *Ps* Lhcas are circled and labelled, differences in carotenoid arrangement between the two structures are indicated. Color codes and PDB ID codes: *Ps* PSI-LHCI, green, 4XK8; *Pp* PSI-LHCI, purple, 6L35.
