# Supplementary material for: Antenna arrangement and energy-transfer pathways of PSI–LHCI from the moss Physcomitrella patens
Source: Cell Discov. 2021 Feb 16;7:10. doi: 10.1038/s41421-021-00242-9 (PMC7884438; doi:10.1038/s41421-021-00242-9)
Supplement: Supplementary file 13 — Fig S13 [file 41421_2021_242_MOESM13_ESM.pdf]

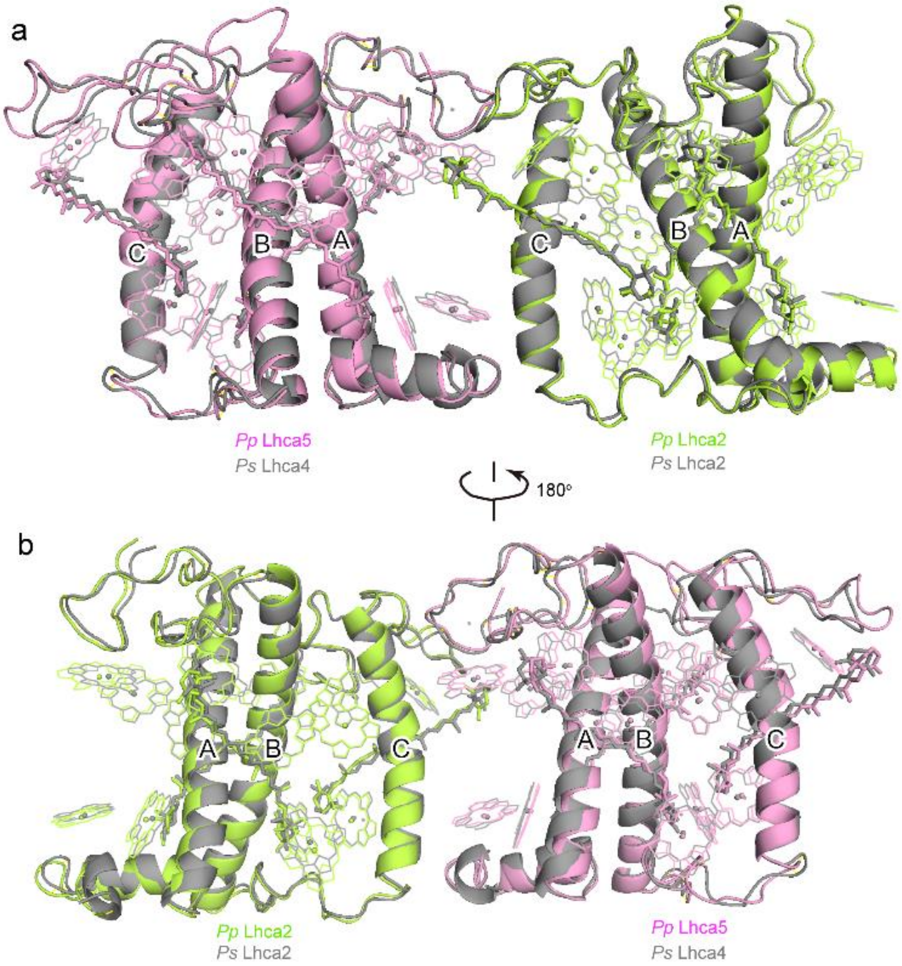

Supplementary Fig. S13 Structural comparison of the *Pp* Lhca5-*Pp* Lhca2 with Lhca4-Lhca2 from higher plants (*P. sativum*). a Superposition of *Pp* Lhca5-Lhca2 with *Ps* Lhca4-Lhca2. View along the membrane plane from the Lhcas side to the PSI core side. **b** View along the membrane plane from the PSI core side to the Lhcas side. Color codes: violet, Lhca5 from *P. patens*; yellow green, Lhca2 from *P. patens*; grey, subunits from *P. sativum.* PDB ID codes: *Pp* PSI-LHCI, 6L35; *Ps* PSI-LHCI, 4XK8.
