# Supplementary material for: Antenna arrangement and energy-transfer pathways of PSI–LHCI from the moss Physcomitrella patens
Source: Cell Discov. 2021 Feb 16;7:10. doi: 10.1038/s41421-021-00242-9 (PMC7884438; doi:10.1038/s41421-021-00242-9)
Supplement: Supplementary file 14 — Table S1 [file 41421_2021_242_MOESM14_ESM.pdf]

**Supplementary Table S1 Cryo-EM data collection and model statistics.**

| | PSI-LHCI (EMBD EMD-30198) | PSI-LHCI (EMBD EMD-0821) (PDB 6L35) |
|---|---|---|
| **Data collection and processing** | | |
| Magnification | 78,000 | 130,000 |
| Voltage (kV) | 200 | 300 |
| camera | Falcon II | K2 |
| Electron exposure ($e^{-}$/Å2) | 50 | 50 |
| Defocus range (μm) | -1.5 ~ -2.5 | -1.5 ~ -2.5 |
| Pixel size (Å) | 1.27 | 1.061 |
| Micrographs (no.) | 4,052 | 6,952 |
| Initial particle images (no.) | 537,999 | 535,146 |
| Final particle images (no.) | 245,039 | 70,288 |
| Symmetry imposed | C1 | C1 |
| Map resolution (Å) | 4.1 | 3.2 |
| FSC threshold | 0.143 | 0.143 |
| Map resolution range (Å) | 10.0-4.1 | 10.0-3.2 |
| **Refinement** | | |
| Initial model used | Initial model generated in RELION3.0 | EMBD |
| Model resolution (Å) | 4.1 | 3.2 |
| FSC threshold | 0.143 | 0.143 |
| Map sharpening *B* factor (Å$^2$) | -274 | -87.6 |
| Model composition | | |
| Non-hydrogen atoms | -- | 34,822 |
| Protein residues | -- | 3,260 |
| Ligands | -- | 205 |
| B factors (Å2) | | |
| Protein | -- | 65.06 |
| Ligand | -- | 61.66 |
| R.m.s. deviations | | |
| Bond lengths (Å) | -- | 0.004 |
| Bond angles (°) | -- | 1.354 |
| Validation | | |
| MolProbity score | -- | 2.59 |
| Clashscore | -- | 10.95 |
| Rotamer outliers (%) | -- | 6.19 |
| Ramachandran plot | | |
| Favored (%) | -- | 93.43 |
| Allowed (%) | -- | 6.19 |
| Disallowed (%) | -- | 0 |
