# Supplementary material for: Antenna arrangement and energy-transfer pathways of PSI–LHCI from the moss Physcomitrella patens
Source: Cell Discov. 2021 Feb 16;7:10. doi: 10.1038/s41421-021-00242-9 (PMC7884438; doi:10.1038/s41421-021-00242-9)
Supplement: Supplementary file 15 — Table S2 [file 41421_2021_242_MOESM15_ESM.pdf]

Supplementary Table S2 Cofactors found in each subunit of the *Pp* PSI-LHCI supercomplex.

Subunit	Possible accession	Traced residues	Chlorophylls	Carotenoids	Lipids	Others
PsaA	NP_904202.1	742 (9-750)	45 Chl *a*	6 BCR	2 LHG	1 SF4<br>1 PQN
PsaB	NP_904203.1	733 (2-734)	40 Chl *a*	7 BCR	1 LHG<br>1 DGD	1 PQN
PsaC	NP_904239.1	80 (2-81)	--	--	--	2 SF4
PsaD	XP_024391925.1<br>XP_024370897.1<br>XP_024379481.1<br>XP_024398188.1	141 (77-217)	--	--	--	--
PsaE	XP_024362597.1<br>XP_024364786.1<br>XP_024358469.1	62 (72-133)	--	--	--	--
PsaF	XP_024402555.1	159 (86-244)	4 Chl *a*	1 BCR	--	--
PsaG	XP_024397215.1	98 (58-155)	3 Chl *a*	1 BCR	--	--
PsaH	XP_024384425.1	90 (51-140)	--	--	--	--
PsaI	NP_904192.1	34 (1-34)	--	1 BCR	--	--
PsaJ	NP_904180.1	41 (1-41)	1 Chl *a*	2 BCR	1 LMG	--
PsaK	XP_024370983.1<br>XP_024370983.1	79 (50-128)	4 Chl *a*	2 BCR	--	--
PsaL	XP_024367813.1	159 (63-221)	3 Chl *a*	2 BCR	--	--
PsaM	NP_904214.1	29 (3-31)	--	--	--	--
Lhca1	XP_024393004.1	192 (50-241)	12 Chl *a*<br>2 Chl *b*	1 XAT<br>1 LUT	1 LHG	--
Lhca5	XP_024372932.1	202 (72-273)	10 Chl *a*<br>4 Chl *b*	1 BCR<br>1 XAT<br>1 LUT	1 LHG	--
Lhca2	XP_024386885.1	206 (63-268)	9 Chl *a*<br>5 Chl *b*	1 BCR<br>1 XAT<br>1 LUT	2 LMG<br>1 LHG	--<br>--
Lhca3	XP_024378501.1<br>XP_024367681.1<br>XP_024367711.1	213 (102-314)	13 Chl.*a*<br>1 Chl *b*	2 BCR<br>1 XAT<br>1 LUT	--	--
Total		3260	156	34	10	5

Abbreviations used: BCR, *β*-carotene; LUT, lutein; XAT, violaxanthins; SF4, iron/ sulfur cluster; PQN, phylloquinone; LHG, 1, 2-dipalmitoyl-phosphatidyl-glycerol; DGD, digalactosyl diacylglycerol; LMG, 1,2-Distearoyl-monogalactosyl diglyceride.
