# Supplementary material for: Antenna arrangement and energy-transfer pathways of PSI–LHCI from the moss Physcomitrella patens
Source: Cell Discov. 2021 Feb 16;7:10. doi: 10.1038/s41421-021-00242-9 (PMC7884438; doi:10.1038/s41421-021-00242-9)
Supplement: Supplementary file 16 — Table S3 [file 41421_2021_242_MOESM16_ESM.pdf]

Supplementary Table S3 Comparison of PSI core subunits from different species.

	Cyano-bacteria	*C. merolae*	*C. reinhardtii*	*B. corticulans*	*P. patens*	*P. sativum & Z. mays*
**PsaA**	√	√	√	√	√	√
**PsaB**	√	√	√	√	√	√
**PsaC**	√	√	√	√	√	√
**PsaD**	√	√	√	√	√	√
**PsaE**	√	√	√	√	√	√
**PsaF**	√	√	√	√	√	√
**PsaG**	-----	-----	√	√	√	√
**PsaH**	-----	-----	√	√	√	√
**PsaI**	√	√	√	√	√	√
**PsaJ**	√	√	√	√	√	√
**PsaK**	√	√	√	√	√	√
**PsaL**	√	√	√	√	√	√
**PsaM**	√	√	±	√	√	-----
**PsaN**	-----	-----	√	√	-----	√
**PsaO**	-----	√	√	√	√	√
**PsaP**	-----	-----	-----	-----	√	√

Annotations: *Cyanobacteria* according to ref. 3; *Cyanidioschyzon merolae* (*C. merolae*) according to ref. 6; *Chlamydomonas reinhardtii* (*C. reinhardtii*) according to ref. 10; *Bryopsis corticulans* (*B. corticulans*) according to ref. 9; *Physcomitrella patens* (*P. patens*) according to ref. 24; *Pisum sativum* (*P. sativum*) according to ref. 12, 13; *Zea mays* (*Z. mays*) according to ref. 14.
